# Identification of Optimal Reference Genes for Gene Expression Normalization in a Wide Cohort of Endometrioid Endometrial Carcinoma Tissues

**DOI:** 10.1371/journal.pone.0113781

**Published:** 2014-12-04

**Authors:** Chiara Romani, Stefano Calza, Paola Todeschini, Renata A. Tassi, Laura Zanotti, Elisabetta Bandiera, Enrico Sartori, Sergio Pecorelli, Antonella Ravaggi, Alessandro D. Santin, Eliana Bignotti

**Affiliations:** 1 “Angelo Nocivelli” Institute of Molecular Medicine, Division of Gynecologic Oncology, University of Brescia, Brescia, Italy; 2 Department of Molecular and Translational Medicine, University of Brescia, Brescia, Italy; 3 Department of Medical Epidemiology and Biostatistics, Karolinska Institutet, Stockholm, Sweden; 4 Department of Obstetrics, Gynecology & Reproductive Sciences, Yale University School of Medicine, New Haven, Connecticut, United States of America; Taipei Medical University, Taiwan

## Abstract

Accurate normalization is a primary component of a reliable gene expression analysis based on qRT-PCR technique. While the use of one or more reference genes as internal controls is commonly accepted as the most appropriate normalization strategy, many qPCR-based published studies still contain data poorly normalized and reference genes arbitrarily chosen irrespective of the particular tissue and the specific experimental design. To date, no validated reference genes have been identified for endometrial cancer tissues. In this study, 10 normalization genes (GAPDH, B2M, ACTB, POLR2A, UBC, PPIA, HPRT1, GUSB, TBP, H3F3A) belonging to different functional and abundance classes in various tissues and used in different studies, were analyzed to determine their applicability. In total, 100 endometrioid endometrial cancer samples, which were carefully balanced according to their tumor grade, and 29 normal endometrial tissues were examined using SYBR Green Real-Time RT-PCR. The expression stability of candidate reference genes was determined and compared by means of geNorm and NormFinder softwares. Both algorithms were in agreement in identifying GAPDH, H3F3A, PPIA, and HPRT1 as the most stably expressed genes, only differing in their ranking order. Analysis performed on the expression levels of all candidate genes confirm HPRT1 and PPIA as the most stably expressed in the study groups regardless of sample type, to be used alone or better in combination. As the stable expression of HPRT1 and PPIA between normal and tumor endometrial samples fulfill the basic requirement of a reference gene to be used for normalization purposes, HPRT1 expression showed significant differences between samples from low-grade and high-grade tumors. In conclusion, our results recommend the use of PPIA as a single reference gene to be considered for improved reliability of normalization in gene expression studies involving endometrial tumor samples at different tumor degrees.

## Introduction

Endometrial carcinoma (EC) is the most common gynecologic malignancy in the Western world, with 52,630 new cases and 8590 deaths expected in the United States alone in 2014 [1]. EC may be broadly divided into two classes, type I and type II, with distinct histopathology, clinical behavior and underlying molecular pathogenesis [2]. Most EC patients have oestrogen-related tumours, well-differentiated, endometrioid in histology and, consequently, with good prognosis (Type I EECs) [3], [4]. In contrast, Type II ECs include serous, clear cell or grade 3 endometrioid histology, which being typified by an aggressive clinical course with a distinct pattern of metastasis, often recur despite aggressive clinical interventions [5], [6].

Gene expression studies have extensively been conducted in cancer research, with the aim of discovering new potential diagnostic and prognostic biomarkers. Quantitative real-time reverse transcription PCR (qRT-PCR) is one of the most powerful and sensitive techniques available for gene expression analysis, with relative quantification as a commonly used strategy for interpreting obtained results. In relative quantification, changes in gene expression in a given sample are expressed relative to another reference sample, after normalization using a stably expressed reference gene simultaneously determined. In this kind of gene expression study, selecting a valid reference gene to correct for differences in RNA sampling is critical to avoid misinterpretation of results [7], [8].

An mRNA used as a valid reference for qRT-PCR ***experiments*** should have the following properties [9]–[12]: constitutive and constant expression in all samples analyzed and similar expression compared to the gene of interest. Moreover, particular attention should be paid to the primer design, since the amplification of the reference gene sequence has to be mRNA specific, without contamination of DNA and pseudogenes.

A ***reference gene often*** adopted from the literature in relative gene expression studies is glyceraldehyde-3-phosphate dehydrogenase (GAPDH), ***a*** common metabolic enzyme that has many functions besides being the most well known involved in the glycolytic pathway. Its levels are not constant and vary more than for other genes across different tissues. Other frequently used reference genes are ribosomal RNAs (28S or 18S), that generally are not an optimal choice, thanks to the combination of (i) high abundance and (ii) different transcription and degradation characteristics compared to mRNAs.

With the aim of finding suitable reference genes for gene expression studies in EC tissue samples, we carried out a Medline search using the MeSH terms “endometrial cancer” and “real-time PCR”. We critically evaluated 327 papers published from May 2000 to February 2014 (see Text S1). We identified a total 102 articles based on the use of Real-Time PCR in gene expression studies on endometrial cancer tissues. Within these reports, we removed papers evaluating microRNA expression and genotyping studies as well as gene expression studies performed on cell cultures or Formalin-Fixed Paraffin Embedded (FFPE) tissues. Twelve additional articles were excluded from analysis because full text was not available in the English language. The remaining 90 articles focusing on gene expression studies in fresh frozen tissues were used in the final analysis. These articles used the following reference genes for data normalization, alone or in combination of two or three genes used in the final data analysis: Glyceraldehyde-3-phosphate dehydrogenase (GAPDH) (39 times), Beta-actin (ACTB) (23 times), 18S-rRNA (22 times), hypoxanthine-guanine phosphoribosyltransferase (HPRT1) (6 times), peptidylprolyl isomerase A (PPIA) (5 times), DNA-directed RNA polymerase II subunit RPB1 (POLR2A) and β-glucuronidase (GUSB) (3 times each), ribosomal protein large P0 (RPLPO), ribosomal protein L19 (L19), and β2-microglobulin (B2M) (2 times each), TATA box binding protein (TBP), ribosomal protein S17 (RPS17), delta-aminolevulinate synthase 1 (ALAS1), HLA-DR antigens-associated invariant chain (CD74), RNase P, cytokeratin 18 (CK18), ubiquitin C (UBC), hydroxymethylbilane synthase (HMBS), importin 8 (IPO8), phosphoglycerate kinase 1 (PGK1), tyrosine 3-monooxygenase/tryptophan 5-monooxygenase activation protein, zeta polypeptide (YWHAZ) and H3 histone, family 3A (H3F3A) (1 time each).

This search highlights that (i) a wide-accepted single or combination of reference genes for endometrial cancer gene expression studies does not currently exist; (ii) ACTB and GAPDH are the most frequently used reference genes for normalization in endometrial cancer research, mostly used as single gene, even if their use for this purpose is controversial; (iii) recently, the Minimum Information for Publication of Quantitative Real-Time PCR Experiments (MIQE) guidelines has shown the importance of using a combination of reference genes for reliable analysis of gene expression data [13] and (iv) a study investigating the most suitable reference genes for endometrial cancer research on a wide cohort of fresh frozen tissues has not been performed until now.

To fill this gap of knowledge, our aim was to extrapolate the most suitable combination of reference genes for qRT-PCR studies from a panel of 10 genes frequently used in the literature in a wide and well-characterized cohort of endometrioid EC and normal endometrium tissue samples.

## Materials and Methods

### Patients and samples

Endometrial cancer tissue samples were obtained from 100 patients (mean age 66 years, range 41–92 years) undergoing total abdominal hysterectomy at the Division of Gynecologic Oncology, University of Brescia. Normal endometrial tissue samples were obtained from 29 patients (mean age 53 years, range 37–79 years) undergoing hysterectomy for benign pathologies (Table 1). The study was performed following the Declaration of Helsinki set of principles and approved by the Research Review Board- the Ethic Committee- of the Spedali Civili, Brescia, Italy (study reference number: 527/B4/4). Written informed consent was obtained from all patients enrolled.

**Table 1:** Clinical and pathologic characteristics of 100 EC and 29 NE patients.

Characteristics	EEC	NE
**n**	100	29
**FIGO stage (%)**		
I	70	
II	11	
III	17	
IV	2	
**Histological Grade (%)**		
G1	30	
G2	33	
G3	37	
**Age at diagnosis (mean years, range)**	66 (41–92)	53 (37–79)

All neoplastic specimens were reviewed in our institution and histological classification was performed according to WHO criteria, while pathological stage was determined according to the International Federation of Gynecologists and Obstetricians (FIGO) standards. All tumors were endometrioid in histology, with different grades of differentiation: 30 out of the 100 tumors were classified as grade G1 (well-differentiated), 33 as G2 (moderately-differentiated), and 37 as G3 (poorly-differentiated) (Table 1).

Specimens were snap-frozen in liquid nitrogen within 30 minutes after surgical removal, and stored at −80°C until further processing.

Only samples containing at least 70% of tumor epithelial cells as assessed by a staff pathologist were used for total RNA extraction. All the normal endometrial samples were verified to be free of any neoplastic pathology before using for total RNA extraction.

### RNA extraction and cDNA synthesis

Total RNA was isolated from tissue samples using TRIZOL reagent (Life Technologies, Inc., Carlsbad, CA) and then further purified using RNeasy Min-elute Clean-up Columns (Qiagen, Valencia, CA), according to the manufacturer's instructions. RNA concentration and 260/280 absorbance ratio (A_260/280_) were measured with Infinite M200 spectrophotometer (Tecan). RNA integrity was assessed with RNA 6000 Nano LabChip kit using the Agilent 2100 Bioanalyzer (Agilent Technologies, Palo Alto, CA, USA) and the RNA integrity number (RIN) generated with Agilent 2100 Expert software [14]. One microgram of total RNA was reverse transcribed using random hexamers in a final volume of 20 µl, according to the SuperScript TM II Reverse Transcriptase protocol (Invitrogen Life Technologies, Carlsbad, CA, USA). All cDNA samples were diluted to 5 ng/µl, dispensed in a 96wells plates and stored at −20°C until use.

### Real-time quantitative PCR (qPCR)

With the aim of identifying the most stable reference genes useful for normalization in qPCR studies on endometrioid endometrial cancer, ten reference genes belonging to different functional classes were evaluated in this study: GAPDH, B2M, ACTB, POLR2A, UBC, PPIA, HPRT1, GUSB, TBP, H3F3A (Table 2). All primer sequences were designed with Beacon Designer software (Premier Biosoft, Palo Alto, CA, USA) and spanned an exon-exon boundary to control for genomic contamination in RNA samples.

**Table 2:** Candidate reference genes selected for evaluation of expression stability.

Gene symbol	GeneBank Accession No.	Gene name	Molecular function	Primer sequence 5′→3′	amplicon size (bp)	PCR efficiency %
GAPDH	NM_002046	Glyceraldehyde-3-phosphate dehydrogenase	glycolitic enzyme	Forward: cccttcattgacctcaactacatg	115	97.65
				Reverse: tgggatttccattgatgacaagc		
B2M	NM_004048	Beta-2-microglobulin	component of the class I MHC	Forward: cattcctgaagctgacagcattc	136	99.25
				Reverse: tgctggatgacgtgagtaaacc		
ACTB	NM_001101	Beta-actin	cytoskeletal structural protein	Forward: cgccgccagctcaccatg	120	93.91
				Reverse: cacgatggaggggaagacgg		
POLR2A	NM_000937	Polymerase (RNA) II (DNA-directed) polypeptide A 220 kDa	DNA-dependent RNA polymerase component	Forward: gagagtccagttcggagtc	84	103.81
				Reverse: gtcgtctctgggtatttgatg		
UBC	NM_021009	Ubiquitin C	involved in DNA repair, protein degradation, cell cycle regulation	Forward: tcgtcacttgacaatgca	120	95.86
				Reverse: atgccttccttatcttggatc		
PPIA	NM_021130	Peptidylprolyl isomerase A (Cyclophilin A)	cyclosporin binding-protein	Forward: gaggaaaaccgtgtactattagc	113	101.03
				Reverse: gggaccttgtctgcaaac		
HPRT1	NM_000194	Hypoxanthine phosphoribosyltransferase 1	involved in the generation of purine nucleotides through the purine salvage pathway	Forward: ctggaaagaatgtcttgattgtg	104	102.1
				Reverse: gaccttgaccatctttggatta		
GUSB	NM_000181	Glucuronidase beta	catalyze breakdown of complex carbohydrates	Forward: atcgccatcaacaacaca	84	102.36
				Reverse: cttgggatacttggaggtg		
TBP	NM_003194	TATA box binding protein	general transcription factor	Forward: ctccactgtatccctccc	118	88.07
				Reverse: ccaagattcactgtggatacaata		
H3F3A	NM_002107	H3 histone, family 3A	nuclear protein	Forward: tgctcaggactttaaaacaga	108	105.67
				Reverse: cacaggttggtgtcttcaa		

A standard curve of five serial dilution points of a representative cDNA sample, ranging from 15 ng to 24 pg, was generated for each assay to determine PCR efficiency according to Rasmussen [15]. The PCR efficiency and correlation coefficients (R^2^) of each primer pair set were generated using the slope of the standard curves and efficiency was calculated by the formula: efficiency (%) = (10^(−1/slope)^−1)×100.

SYBR green real-time PCR was performed with CFX96 Real-Time system and iTaq Universal SYBR Green Supermix (BIO-RAD Laboratories, Hercules, CA, USA). Amplification mixture (20 µl) contained 15 ng of template cDNA, 2× Mix and 400 nM forward and reverse primers optimized to give maximum amplification efficiency while minimizing nonspecific amplification, with the exception of GUSB whose primers final concentration in reaction was 250 nM.

The cycle conditions were set as follow: 98°C for 30 s, (95°C for 5 s, 60°C for 20 s)×40 cycles. All reactions were run in triplicate and no template controls (no cDNA in PCR) were included in each assay run for each gene. A melting curve was constructed for each primer pair to confirm amplification product specificity.

An inter-run calibration sample was used in all plates to correct for the technical variance between the different runs and to compare results from different plates.

For the quantitative comparison of investigated candidate reference gene amplification rates, Cq (quantification cycle) values were used, according to the RDML (Real-Time PCR Data Markup Language) data standard [16].

### Statistical analysis

Stability of candidate reference genes was calculated with freely available geNorm [11] and NormFinder [17] softwares. Both algorithms identify the most stable control gene in a given set of tissue samples and determine the optimal number of reference genes required for reliable normalization of qPCR data. Differences in gene expression between groups were tested using linear models on log_10_ transformed gene expression values, with p-values and confidence intervals estimation based on “White-Huber” heteroscedasticity corrected covariance matrices [18]. To account for the presence of potential outliers we fitted weighted least squares with weights computed by M-estimation [19]. To test non-difference of gene expression among groups we used the Two-One-Sided Test (TOST) approach, a type of intersection union test [20]. Briefly an “equivalence range” [*ε*_L_,*ε*_U_] is defined. The null hypothesis is set up so that if the 90% confidence interval for the parameter of interest (e.g. the difference among group means) falls completely within the equivalence range, the null hypothesis can be rejected. Two one-sided tests were conducted for both boundaries of the range. The overall null hypothesis is rejected at level α if the associated p-value for each of the individual hypotheses is less than α (α = 0.05 in our analysis). When the test analysed more than two groups we considered all possible pairwise contrasts confidence intervals. In such cases the null of no equivalence was not rejected if at least one of them didn't fall within the equivalence range. All models included age as a covariate.

To conduct an equivalence test, one must choose, prior to conducting the test, an equivalence range i.e., the range in which we can consider the parameter of interest in the two groups to be substantively equal. While no fixed objective rules exist to guide the choice of the equivalence range because such choice may depend on substantive considerations, Wellek [21] suggested a strict tolerance value for a two sample t-test of ±0.36. In contrast, the Food and Drug Administration (FDA) requires a ±20% rule: (i.e., bioequivalence is accepted if the mean bioavailability of the test formulation is within ±20% of the mean of the reference formulation) [22]. However, for a logarithmic transformation of the responses, the FDA guidance requests that to claim average bioequivalence, the ratio of the two groups averages on the original scale be within the range [0.8–1.25] (±0.2231 on log scale). Because gene expression is routinely analysed on log scale we adopted this latest criteria in our data analysis.

Statistical analysis were performed using **R** [23] with addititional package *robustbase* [24] (Text S2).

## Results

### RNA quality

RNA samples isolated from 100 malignant and 29 normal endometrial specimens were examined for concentration, purity and integrity. RNA samples A260/280 ratio ranged from 1.77 to 2.16 (mean 1.98± SD 0.09), reflecting pure and protein-free isolated RNA. The integrity was assessed by RIN values using the Agilent 2100 Bioanalyzer. Four RNAs from normal endometrial and 8 from EC tissues were excluded from further gene expression studies due to their high degradation (RIN<5). Mean RIN values of remaining malignant and non-malignant samples were acceptable and not significantly different (7.36±1.54 and 7.55±1.09 respectively, p = 0.59).

### Expression levels of candidate reference genes

The Cq (quantification cycle) values of 10 candidate reference genes were converted to CNRQ (calibrated normalized relative quantities) values using qBase software (http://medgen.ugent.be/qBase), according to Hellemans et al [25]. This normalization procedure removes the variation resulting from the use of different cDNA concentration and correct for run-to-run variation using an internal control gene.

**Figure 1:**
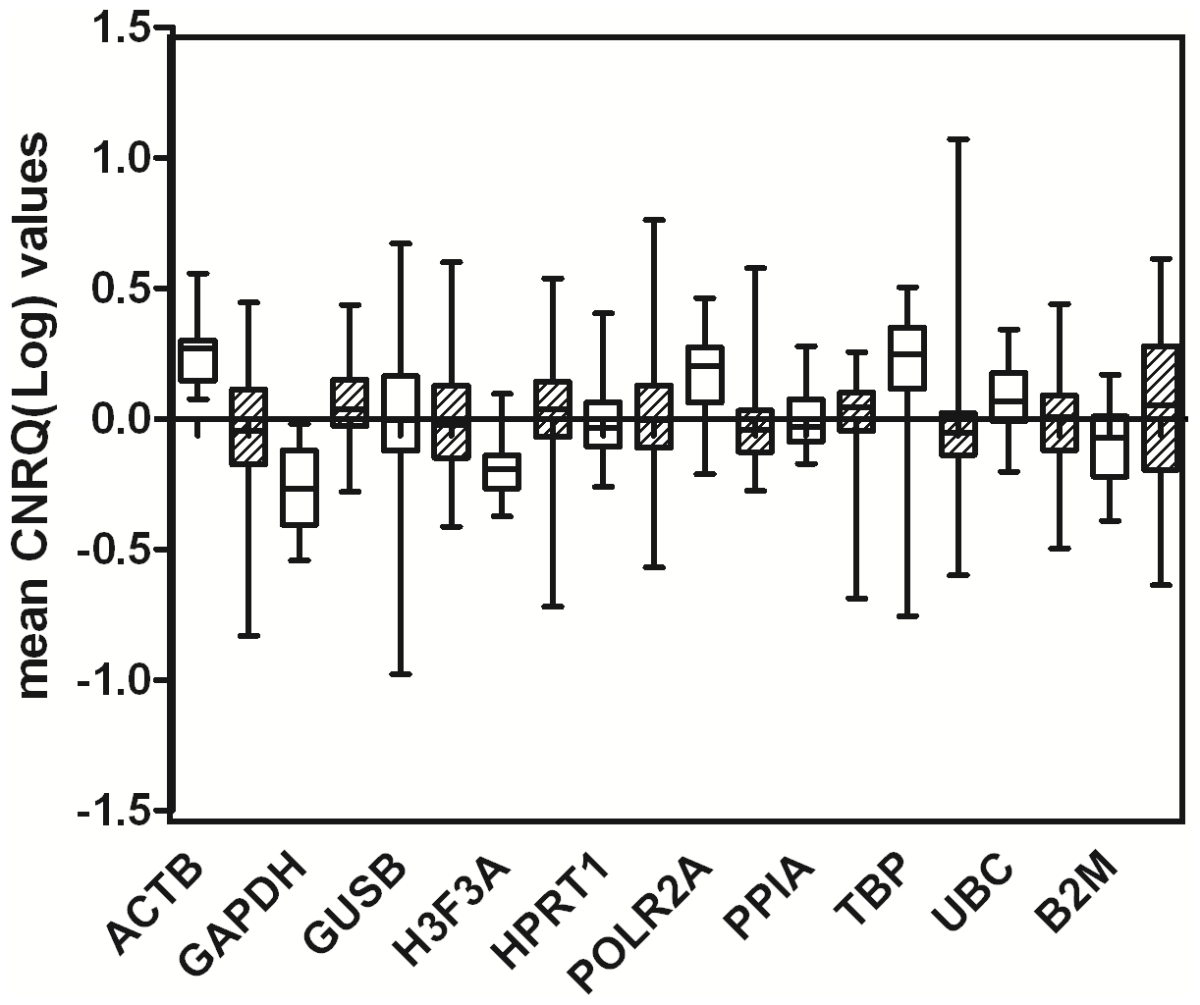
Expression levels of candidate reference genes in malignant (hatched boxes) and non-malignat (open boxes) endometrial samples. Values are given as calibrated normalized relative quantities (CNRQ). Boxes indicate IQR for the data of unmatched samples in each group.

Data on non-malignant and malignant samples are separately shown as box plot in Fig. 1. All reference genes showed a substantial higher spread in malignant samples compared to non-malignant ones. The genes showing the higher Interquatile-range (IQR) were B2M (IQR = 0.402) and ACTB (IQR = 0.295) while PPIA, POLR2A and HPRT1 show the smallest IQR (0.153, 0.187 and 0.197 respectively).

Applying the TOST method equates at computing (1–2α)% confidence interval (90% when α = 0.05) for the contrast of interest (Δ = difference between group means) and comparing it to the [*ε*_L_,*ε*_U_] range, if the Confidence Interval (CI) intersects the equivalence range on either side the null hypothesis of non equivalence can be rejected. Table 3 shows 90% confidence intervals for all the contrasts of interests and the ANOVA p-values as well as an indicator of null hypothesis rejection based on an estimate of *ε* = 0.015. Considering the difference in gene expression between malignant and non-malignant samples, all genes were substantially varying but for GUSB, HPRT1 and PPIA (Table 3).

**Table 3:** Fold changes in reference gene expression between malignant and non-malignant endometrial samples and among malignant samples grouped according to tumor grade and stage (90% confidence intevals, Lower and Upper bounderies; ^$^ p-value for one-way ANOVA; R = * null hypothesis of non equivalence rejected; [ε_L_,ε_U_] = [0.8,1.25]).

	EC vs NE				GRADE								STAGE			
					G2 vs G1		G3 vs G1		G3 vs G2				III–IV vs I–II			
Gene	Lower	Upper	R	p-value^$^	Lower	Upper	Lower	Upper	Lower	Upper	R	p-value^$^	Lower	Upper	R	p-value^$^
ACTB	0.50	0.65		<0.001	0.86	1.16	0.97	1.59	0.99	1.55		0.273	0.48	1.56		0.677
GAPDH	1.83	2.49		<0.001	0.71	1.02	0.71	0.96	0.84	1.11		0.099	1.03	1.49		0.057
GUSB	0.75	1.15		0.560	0.61	0.87	0.60	0.88	0.84	1.19		0.007	0.79	1.26		0.973
PPIA	0.99	1.25		0.137	0.95	1.14	1.01	1.24	0.97	1.19	*	0.188	0.85	1.14	*	0.880
HPRT1	0.83	1.09	*	0.530	1.28	1.69	1.34	1.79	0.91	1.22		<0.001	0.98	1.49		0.147
H3F3A	1.48	1.85		<0.001	1.07	1.45	0.98	1.32	0.77	1.09		0.057	0.81	1.13	*	0.675
POLR2A	0.51	0.69		<0.001	0.78	0.95	0.92	1.15	1.08	1.34		0.007	0.94	1.18	*	0.485
TBP	0.40	0.50		<0.001	1.01	1.26	0.92	1.18	0.81	1.05		0.167	0.98	1.39		0.142
UBC	0.73	0.93		0.012	1.11	1.54	1.07	1.48	0.84	1.12		0.019	0.92	1.28		0.402
B2M	1.22	1.87		0.002	0.52	0.94	0.62	1.05	0.84	1.59		0.114	0.71	1.40		0.996

Models adjusted for age.

Among malignant samples, the expression of reference genes was substantially equivalent among tumor grade groups for ACTB, PPIA and TBP. Considering groups based on tumor stage the average expression of all genes except for GAPDH was substantially stable (Table 3).

### Expression stability of candidate reference genes

**Figure 2:**
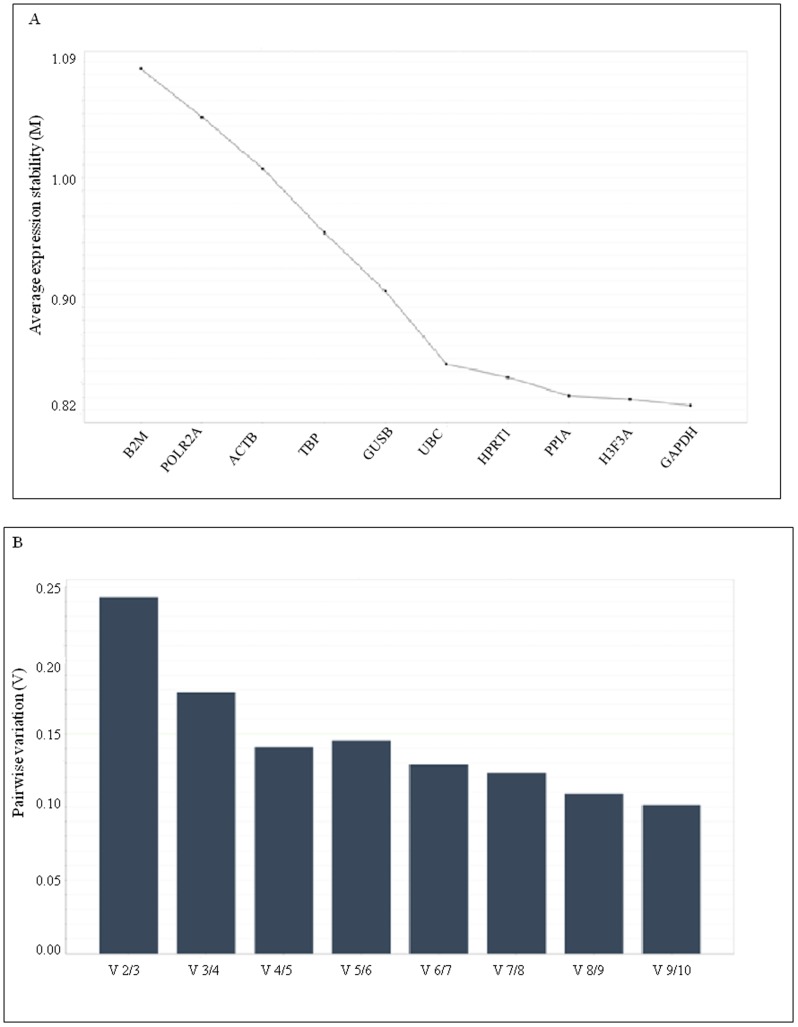
Selection of the most suitable reference genes for normalization in endometrial cancer samples using geNorm analysis. Results are presented according to the output file of the geNorm program [11]. (A) Average expression stability value (M) of control genes calculated by a stepwise exclusion of the least stable genes: the program enables elimination of the worst-scoring gene with the highest M value and recalculation of new M value for the remaining genes. X axis from the left to the right indicates gene rank according to expression stability, while Y axis indicate the stability measure M. (B) Determination of the optimal number of control genes for normalization.

From the theoretical point of view, genes whose expression are equivalent between malignant and non malignant tissue samples are all suitable reference for relative quantification of target genes in gene profiling studies. Thus, according to the statistical analysis performed on the expression level of the 10 selected reference genes, GUSB, HPRT1 and PPIA were the best candidate for normalization purposes in gene expression studies in endometrial cancer versus normal tissue. To validate and confirm this result, all 10 reference genes including the best performing GUSB, HPRT1 and PPIA were included in the program geNorm which calculated the gene expression stability measure M of one gene based on the average pairwise variation between all studied genes. The lowest M value characterized the gene with the most stable expression. As shown in Fig. 2A, all genes studied achieved medium expression stability with M values ranging from 1.09 for B2M to 0.82 for GAPDH (average geNorm M≤1.0), which is typically seen when evaluating candidate reference targets on heterogeneous samples, like cancer biopsies or samples from different tissues. GAPDH, H3F3A, PPIA and HPRT1 were identified as the most stable genes, followed by UBC as the fifth most stable gene. Furthermore, in addition to the generated M stability value, the geNorm program calculates a normalization factor, based on the variable V as the pairwise variation between two sequential normalization, to assess the optimal number of genes required for a reliable normalization of qPCR data. As shown in fig. 2B, taking 0.15 as a cut-off value below which the inclusion of an additional control gene is not required [11], the optimal number of reference targets was 4. As such, based on geNorm analysis, in our experimental situation the optimal normalization factor could be calculated as the geometric mean of reference targets HPRT1, PPIA, H3F3A and GAPDH. Additionally, we compared and validated the results generated from geNorm using the NormFinder program, that selected UBC and PPIA with a stability value of 0.365 and 0.370 respectively as the most stable genes, followed by HPRT1, H3F3A and GAPDH closely behind (Table 4). GeNorm and Normfinder results are in considerably closer agreement in the identification of B2M, ACTB and POLR2A as the least stable genes in our cohort of samples.

**Table 4:** Candidate reference genes for normalization listed according to their expression stability calculated by the NormFinder program.

Ranking order	Gene name	Stability value
1	UBC	0.365
2	PPIA	0.370
3	HPRT1	0.436
4	H3F3A	0.473
5	GAPDH	0.480
6	TBP	0.566
7	GUSB	0.586
8	POLR2A	0.653
9	ACTB	0.657
10	B2M	0.682

High expression stability is indicated by a low stability value as an estimation of both the intra- and the intergroup variation of individual gene, according to the model-based approach developed by Andersen et al. [17].

## Discussion

Selection of appropriate reference genes is a critical aspect to be considered for interpretation of qPCR data [13]. Normalization is a primary component of a reliable gene expression analysis, because this process controls for biological and analytical variation characterized by the natural variability of mRNA levels in different tissues and individuals, as well as differences in RNA quality, extraction protocols and PCR efficiency [26]. The use of reference genes as internal controls is currently the widely used method for normalizing mRNA data and correcting them for cellular and experimental variability. Furthermore, it has become well-estabilished that the use of more than one reference gene increases the accuracy of the measurement compared to a single reference gene, with the mean expression value as the best normalization strategy [13].

Nevertheless, the vast majority of qPCR-based published studies reveals an insufficient and inadequate validation of endogenous control genes, often arbitrarily chosen among those most commonly published and almost always used as single reference gene and not in combination [27].

To the best of our knowledge, no validated reference genes have been identified for EC tissues. A comprehensive MEDLINE search of gene expression studies published between 2000 and the beginning of 2014 showed that there was no uniform opinion on which reference gene should be used for reliable normalization in EC samples. Only one article about reference gene evaluation in EC tissue samples is reported by Hevir N et al [28]. In that investigation 38 endometrial tumors and associated normal specimens were examined using qPCR, followed by GeNorm and NormFinder algorithms, that found HPRT1, POLR2A and PPIA as the most stable reference genes. However, that study was performed using RNA samples obtained from a small cohort of tumor and normal endometrial tissues. Furthermore, tumor histological type was not specified and the percentage of tumor cells in the samples was not addressed.

According to our literature analysis, GAPDH, 18S rRNA and ACTB were the most frequently reported control genes, although their usefulness as normalizers was not always strictly evaluated. Moreover, 18S rRNA have already been described as a poor choice for use as a reference gene, since its transcription is significantly regulated in various experimental settings and variable in different tissues. Indeed, the regulation of ribosomal RNA synthesis is independent from mRNA synthesis, resulting in a different expression pattern compared to mRNA [12].

The current study offers a systematic evaluation of 10 potential reference genes in a large set of endometrioid EC samples and normal endometrial tissues, to determine the most reliable one for accurate normalization of RT-qPCR data. We examined, in addition to GAPDH and ACTB, eight other genes (GUSB, H3F3A, HPRT1, POLR2A, PPIA, TBP, UBC, B2M) that have been evaluated as reference genes in previously published EC gene expression studies.

The particular design of our study has been characterized by several features, including 1) wide cohort of malignant and non-malignant endometrial samples, belonging to a single institution, 2) tumor tissue samples of endometrioid histological type, the most frequent EC type, and number well balanced with respect to tumor grade, 3) stringent quality control of isolated total RNA, 4) careful selection of reference genes, avoiding rRNA and genes whose proteins belong to similar functional classes, to reduce gene coregulation, 5) optimal primer set design of selected reference genes, each one validated with a dissociation curve of the amplicon, 6) qPCR performed with SYBR green technology, that is cost-effective and easy to apply in every laboratory setting, 7) use of an inter-run calibration sample, that showed an optimal correlation in gene expression among all plates, 8) experiments performed in triplicate for every gene and every sample.

In particular, choosing optimal primer set is an essential starting point to obtain accurate results. We adopted primer sets generating amplicons with length around 100 base pair, in order to obtain the maximum amplification efficiency, independent of RNA quality, as previously reported [29]. Moreover, all primer sets were required to span at least two neighboring exons, to avoid amplified products from contaminating genomic DNA eventually present, that can also affect amplification efficiency [30]. To determine the best reference genes, we analyzed our results under the suggested published rules [31]. First, considering amplification efficiency, all primer sets are acceptable because they show a performance close to 100% (Table 2). Then, we calculated the best performing reference genes using geNorm and NormFinder softwares, based on two distinct statistical models and often used in other studies to identify suitable reference genes from a set of candidates [11], [17]. While geNorm indicated GAPDH, H3F3A, PPIA and HPRT1 as the four most stably expressed reference genes, NormFinder identified UBC and PPIA, strictly followed by HPRT1 and H3F3A, as the four genes with the best stability. However, our previously performed statistical analysis on gene expression of our cohort of samples, revealed that only GUSB, HPRT1 and PPIA did not differ significantly in their expression in malignant versus non-malignant endometrial tissues. Conversely, GAPDH and ACTB, the two most commonly reported normalization genes, did not fulfill the criteria of constant expression between normal and tumor samples, as already described by several authors for other malignancies [25], [32].

A potential limitation of this study is represented by the discrepancy in the number of cases (#100) analyzed when compared to healthy controls (#29). The study is however characterized by a powerful parametric approach which partially corrects for this unbalance. In this context, it is not advisable to blindly accept the best combination suggested by geNorm and NormFinder, as both algorithms included genes like GAPDH and H3F3A showing differences in expression level between normal and tumor endometrial tissues. Actually, combining the results from the powerful statistical analysis and the expression stability performed on the expression level of 10 candidate reference genes, HPRT1 and PPIA consistently emerged as the most stable expressed genes, regardless of sample type, to be used as normalizer for relative gene quantification in endometrioid EC samples versus normal controls. These results, common to geNorm and NormFinder, are in concordance with previously published data on a smaller cohort of EC samples [26]. Moreover HPRT1 has previously been recommended as a universal reference gene for differential expression studies in cancer research [33].

However, considering that the expression of reference genes can be influenced not only by the tissue type, but also by other clinico-pathological characteristics like tumor grade or stage, to prove whether a gene is suitable for normalization it is necessary to carefully define the problem to be investigated. It must be decided, for instance, whether gene expression only between normal and tumor samples, or also among different tumor grades and/or stages has to be compared, taking into account that the basic requirement of a reference gene to be used for normalization purposes is that its expression does not show significant difference between the study groups. The results of our analysis proved that the expression of reference genes in our cohort of patients, including only endometrioid histotype and carefully selected and balanced according to their tumor grade, was dependent on tumor grade for B2M, GAPDH, H3F3A, GUSB, HPRT1, POLR2A and UBC. Among the remaining genes, PPIA resulted as the best performing reference gene to be used for normalization of endometrial carcinomas at different degrees of differentiation. This result was first achieved using a powerful parametric statistical approach, followed by a validation using geNorm and NormFinder softwares.

Differently, expression of all genes except for GAPDH shows to remain stable among tumor stages, but the extreme imbalance in the number of patients per group has to be considered in the evaluation of those results.

In summary, careful selection of appropriate endogenous control genes plays a crucial role in expression studies. The current investigation reports the first evaluation of a panel of putative reference genes in endometrioid EC tissue samples, the most frequent EC histotype. Taking our findings together, we recommend HPRT1 and PPIA as the best reference genes for relative quantification in gene expression studies comparing normal and tumor endometrial tissues. They can be used as single reference genes depending on the expression level of the target gene, or better in combination to achieve a more reliable normalization strategy. Finally, this is the first study exploring reference gene expression in endometrioid EC tissues among the different tumor degrees of differentiation, where PPIA has emerged as the most appropriate single reference gene for accurate data normalization.

## Supporting Information

Text S1 **Complete list of references (i.e., 327 papers) cited in the Background section of the manuscript.** The 90 articles based on the use of one or more reference genes in endometrial cancer expression studies and used in our statistical analysis are highlighted in bold. Twelve articles (highlighted in italics in the text) were excluded from analysys because not available in the English language.
(DOC)

Text S2 **Algorithm for testing equivalence and R code examples.**
(DOC)
