# Supplementary material for: Identification of Optimal Reference Genes for Gene Expression Normalization in a Wide Cohort of Endometrioid Endometrial Carcinoma Tissues
Source: PLoS One. 2014 Dec 4;9(12):e113781. doi: 10.1371/journal.pone.0113781 (PMC4256201; doi:10.1371/journal.pone.0113781)
Supplement: Text S1 — Complete list of references (i.e., 327 papers) cited in the Background section of the manuscript. The 90 articles based on the use of one or more reference genes in endometrial cancer expression studies and used in our statistical analysis are highlighted in bold. Twelve articles (highlighted in italics in the text) were excluded from analysys because not available in the English language. (DOC) [file pone.0113781.s001.doc]

**Text S1:** Complete list of references (i.e., 327 papers) cited in the Background section of the manuscript. The 90 articles based on the use of one or more reference genes in endometrial cancer expression studies and used in our statistical analysis are highlighted in bold. Twelve articles (highlighted in italics in the text) were excluded from analysys because not available in the English language.

1. **Chromatin composition alterations and the critical role of MeCP2 for epigenetic silencing of progesterone receptor-B gene in endometrial cancers. Chu Y, Wang Y, Zhang G, Chen H, Dowdy SC, Xiong Y, Liu F, Zhang R, Li J, Jiang SW. Cell Mol Life Sci. 2014 Sep;71(17):3393-408. PMID:**
2. **Comparative assessment of lymph node micrometastasis in cervical, endometrial and vulvar cancer: insights on the real time qRT-PCR approach versus immunohistochemistry, employing dual molecular markers. Pappa KI, Rodolakis A, Christodoulou I, Gazouli M, Markaki S, Antsaklis A, Anagnou NP. Biomed Res Int. 2014;2014:187684.**
3. Novel three-dimensional in vitro models of ovarian endometriosis. Brueggmann D, Templeman C, Starzinski-Powitz A, Rao NP, Gayther SA, Lawrenson K. J Ovarian Res. 2014 Feb 6;7(1):17.
4. *[Expression of E-cadherin and beta1-integrin mRNA in endometrial cancer]. Wójcik-Krowiranda K, Forma E, Zaczek A, Bryś M, Anna MK, Bieńkiewicz A. Ginekol Pol. 2013 Nov;84(11):910-4.*
5. *[Study of claudin-4 in the diagnosis and treatment of endometrial carcinoma]. Pan XY, Li X, Che YC, Li X, Zhang Y. Zhonghua Fu Chan Ke Za Zhi. 2013 Oct;48(10):768-71. Chinese.*
6. Tubal origin of ovarian endometriosis. Yuan Z, Wang L, Wang Y, Zhang T, Li L, Cragun JM, Chambers SK, Kong B, Zheng W. Mod Pathol. 2014 Aug;27(8):1154-62.
7. **Gene expression changes after ionizing radiation in endothelial cells derived from human endometrial cancer-preliminary outcomes. Liu T, Du X, Sheng X. Arch Gynecol Obstet. 2014 Jun;289(6):1315-23.**
8. Aberrant microRNA expression in endometrial carcinoma using formalin-fixed paraffin-embedded (FFPE) tissues. Lee TS, Jeon HW, Kim YB, Kim YA, Kim MA, Kang SB. PLoS One. 2013 Dec 9;8(12):e81421.
9. Activation of a positive feedback loop involving IL-6 and aromatase promotes intratumoral 17β-estradiol biosynthesis in endometrial carcinoma microenvironment. Che Q, Liu BY, Liao Y, Zhang HJ, Yang TT, He YY, Xia YH, Lu W, He XY, Chen Z, Wang FY, Wan XP. Int J Cancer. 2014 Jul 15;135(2):282-94.
10. **Topoisomerase IIβ binding protein 1 c.*229C&gt;T (rs115160714) gene polymorphism and endometrial cancer risk. Forma E, Wójcik-Krowiranda K, Jóźwiak P, Szymczyk A, Bieńkiewicz A, Bryś M, Krześlak A. Pathol Oncol Res. 2014 Jul;20(3):597-602.**
11. The occurrence of fetal microchimeric cells in endometrial tissues is a very common phenomenon in benign uterine disorders, and the lower prevalence of fetal microchimerism is associated with better uterine cancer prognoses. Hromadnikova I, Kotlabova K, Pirkova P, Libalova P, Vernerova Z, Svoboda B, Kucera E. DNA Cell Biol. 2014 Jan;33(1):40-8.
12. **HE4 transcription- and splice variants-specific expression in endometrial cancer and correlation with patient survival. Jiang SW, Chen H, Dowdy S, Fu A, Attewell J, Kalogera E, Drapkin R, Podratz K, Broaddus R, Li J. Int J Mol Sci. 2013 Nov 18;14(11):22655-77.**
13. Effects of progesterone and its metabolites on human granulosa cells. Pietrowski D, Gong Y, Mairhofer M, Gessele R, Sator M. Horm Metab Res. 2014 Feb;46(2):133-7.

# Extracellular signal-regulated kinase 1/2 signaling pathway is required for **endometrial** decidualization in mice and human. [**Lee CH**](http://www.ncbi.nlm.nih.gov/pubmed?term=Lee CH%5BAuthor%5D&cauthor=true&cauthor_uid=24086495)^1^, [**Kim TH**](http://www.ncbi.nlm.nih.gov/pubmed?term=Kim TH%5BAuthor%5D&cauthor=true&cauthor_uid=24086495), [**Lee JH**](http://www.ncbi.nlm.nih.gov/pubmed?term=Lee JH%5BAuthor%5D&cauthor=true&cauthor_uid=24086495), [**Oh SJ**](http://www.ncbi.nlm.nih.gov/pubmed?term=Oh SJ%5BAuthor%5D&cauthor=true&cauthor_uid=24086495), [**Yoo JY**](http://www.ncbi.nlm.nih.gov/pubmed?term=Yoo JY%5BAuthor%5D&cauthor=true&cauthor_uid=24086495), [**Kwon HS**](http://www.ncbi.nlm.nih.gov/pubmed?term=Kwon HS%5BAuthor%5D&cauthor=true&cauthor_uid=24086495), [**Kim YI**](http://www.ncbi.nlm.nih.gov/pubmed?term=Kim YI%5BAuthor%5D&cauthor=true&cauthor_uid=24086495), [**Ferguson SD**](http://www.ncbi.nlm.nih.gov/pubmed?term=Ferguson SD%5BAuthor%5D&cauthor=true&cauthor_uid=24086495), [**Ahn JY**](http://www.ncbi.nlm.nih.gov/pubmed?term=Ahn JY%5BAuthor%5D&cauthor=true&cauthor_uid=24086495), [**Ku BJ**](http://www.ncbi.nlm.nih.gov/pubmed?term=Ku BJ%5BAuthor%5D&cauthor=true&cauthor_uid=24086495), [**Fazleabas AT**](http://www.ncbi.nlm.nih.gov/pubmed?term=Fazleabas AT%5BAuthor%5D&cauthor=true&cauthor_uid=24086495), [**Lim JM**](http://www.ncbi.nlm.nih.gov/pubmed?term=Lim JM%5BAuthor%5D&cauthor=true&cauthor_uid=24086495), [**Jeong JW**](http://www.ncbi.nlm.nih.gov/pubmed?term=Jeong JW%5BAuthor%5D&cauthor=true&cauthor_uid=24086495). PLOS ONE 2013 Sept 24; 8(9).

1. Down-regulation of miR-145 and miR-143 might be associated with DNA methyltransferase 3B overexpression and worse prognosis in endometrioid carcinomas. Zhang X, Dong Y, Ti H, Zhao J, Wang Y, Li T, Zhang B. Hum Pathol. 2013 Nov;44(11):2571-80.
2. PCAF impairs endometrial receptivity and embryo implantation by down-regulating β3-integrin expression via HOXA10 acetylation. Zhu LH, Sun LH, Hu YL, Jiang Y, Liu HY, Shen XY, Jin XY, Zhen X, Sun HX, Yan GJ. J Clin Endocrinol Metab. 2013 Nov;98(11):4417-28.
3. **Evaluation of expression of the PTEN gene, oestrogen and progesterone receptors as diagnostic and predictive factors in endometrial cancer. Samulak D, Grosman-Dziewiszek P, Michalska MM, Mojs E, Samulak K, Romanowicz H, Smolarz B. Pathol Oncol Res. 2014 Jan;20(1):191-6.**
4. TGFβ-pathway is down-regulated in a uterine carcinosarcoma: a case study. Semczuk A, Zakrzewski PK, Forma E, Cygankiewicz AI, Semczuk-Sikora A, Bryś M, Rechberger T, Krajewska WM. Pathol Res Pract. 2013 Nov;209(11):740-4.
5. **Altered expression of ERs, aromatase, and COX2 connected to estrogen action in type 1 endometrial cancer biology. Jarzabek K, Koda M, Walentowicz-Sadlecka M, Grabiec M, Laudanski P, Wolczynski S. Tumour Biol. 2013 Dec;34(6):4007-16.**
6. The evaluation of the FOXO1, KLF9 and YT521 genes expression in human endometrial cancer. Korani M, Fallah S, Tehranian A, Nourbakhsh M, Samadikuchaksaraei A, Pour MS, Maleki J. Clin Lab. 2013;59(5-6):483-9.
7. CAPN 7 promotes the migration and invasion of human endometrial stromal cell by regulating matrix metalloproteinase 2 activity. Liu H, Jiang Y, Jin X, Zhu L, Shen X, Zhang Q, Wang B, Wang J, Hu Y, Yan G, Sun H. Reprod Biol Endocrinol. 2013 Jul 15;11:64.
8. MicroRNA-199a-3p regulates endometrial cancer cell proliferation by targeting mammalian target of rapamycin (mTOR). Wu D, Huang HJ, He CN, Wang KY. Int J Gynecol Cancer. 2013 Sep;23(7):1191-7.
9. **Treatment with Tie2-siRNA in combination with carboplatin suppresses the growth of Ishikawa human endometrial carcinoma cell xenografts <i>in vivo.</i> Guo F, Xun Q, Zhou H. Oncol Lett. 2013 Jun;5(6):1777-1782. Epub 2013 Apr 8.**
10. Selection and validation of endogenous controls for microRNA expression studies in endometrioid endometrial cancer tissues. Torres A, Torres K, Wdowiak P, Paszkowski T, Maciejewski R. Gynecol Oncol. 2013 Sep;130(3):588-94.
11. Inhibition of enhancer of zeste homolog 2 (EZH2) expression is associated with decreased tumor cell proliferation, migration, and invasion in endometrial cancer cell lines. Eskander RN, Ji T, Huynh B, Wardeh R, Randall LM, Hoang B. Int J Gynecol Cancer. 2013 Jul;23(6):997-1005.
12. p21-activated kinase 4 regulation of endometrial cancer cell migration and invasion involves the ERK1/2 pathway mediated MMP-2 secretion. Lu W, Xia YH, Qu JJ, He YY, Li BL, Lu C, Luo X, Wan XP. Neoplasma. 2013;60(5):493-503.
13. **Hypomethylation of ETS transcription factor binding sites and upregulation of PARP1 expression in endometrial cancer. Bi FF, Li D, Yang Q. Biomed Res Int. 2013;2013:946268.**
14. Estrogen and estrogen receptor induce matrix metalloproteinase-26 expression in endometrial carcinoma cells. Nishi H, Kuroda M, Isaka K. Oncol Rep. 2013 Aug;30(2):751-6.
15. Alterations in expression pattern of splicing factors in epithelial ovarian cancer and its clinical impact. Iborra S, Hirschfeld M, Jaeger M, Zur Hausen A, Braicu I, Sehouli J, Gitsch G, Stickeler E. Int J Gynecol Cancer. 2013 Jul;23(6):990-6.
16. Silencing of ghrelin receptor expression inhibits endometrial cancer cell growth in vitro and in vivo. Fung JN, Jeffery PL, Lee JD, Seim I, Roche D, Obermair A, Chopin LK, Chen C. Am J Physiol Endocrinol Metab. 2013 Jul 15;305(2):E305-13.
17. Expression of DJ-1 in endometrial cancer: close correlation with clinicopathological features and apoptosis. Shu K, Xiao Z, Long S, Yan J, Yu X, Zhu Q, Mei T. Int J Gynecol Cancer. 2013 Jul;23(6):1029-35.
18. GALR1 methylation in vaginal swabs is highly accurate in identifying women with endometrial cancer. Doufekas K, Hadwin R, Kandimalla R, Jones A, Mould T, Crowe S, Olaitan A, Macdonald N, Fiegl H, Wik E, Salvesen HB, Widschwendter M. Int J Gynecol Cancer. 2013 Jul;23(6):1050-5.
19. Bortezomib induces apoptosis of endometrial cancer cells through microRNA-17-5p by targeting p21. Shen Y, Lu L, Xu J, Meng W, Qing Y, Liu Y, Zhang B, Hu H. Cell Biol Int. 2013 Oct;37(10):1114-21.
20. **Silencing of Cathepsin B suppresses the proliferation and invasion of endometrial cancer. Bao W, Fan Q, Luo X, Cheng WW, Wang YD, Li ZN, Chen XL, Wu D. Oncol Rep. 2013 Aug;30(2):723-30.**
21. **VEGF-c expression in an in vivo model of orthotopic endometrial cancer and retroperitoneal lymph node metastasis. Huang YW, Xu LQ, Luo RZ, Huang X, Hou T, Zhang YN. Reprod Biol Endocrinol. 2013 May 21;11:49.**
22. **Overexpression of claudin-4 may be involved in endometrial tumorigenesis. Pan XY, Li X, Che YC, Li HY, Li X, Zhang Y, Yang X. Oncol Lett. 2013 Apr;5(4):1422-1426.**
23. Utilization of genomic signatures to identify high-efficacy candidate drugs for chemorefractory endometrial cancers. Kharma B, Baba T, Mandai M, Matsumura N, Murphy SK, Kang HS, Yamanoi K, Hamanishi J, Yamaguchi K, Yoshioka Y, Konishi I. Int J Cancer. 2013 Nov;133(9):2234-44.
24. Combination of Vorinostat and caspase-8 inhibition exhibits high anti-tumoral activity on endometrial cancer cells. Bergadà L, Sorolla A, Yeramian A, Eritja N, Mirantes C, Matias-Guiu X, Dolcet X. Mol Oncol. 2013 Aug;7(4):763-75.
25. Tubulin-β-III overexpression by uterine serous carcinomas is a marker for poor overall survival after platinum/taxane chemotherapy and sensitivity to epothilones. Roque DM, Bellone S, English DP, Buza N, Cocco E, Gasparrini S, Bortolomai I, Ratner E, Silasi DA, Azodi M, Rutherford TJ, Schwartz PE, Santin AD. Cancer. 2013 Jul 15;119(14):2582-92.
26. Clinical significance of galectin-7 in epithelial ovarian cancer. Kim HJ, Jeon HK, Lee JK, Sung CO, Do IG, Choi CH, Kim TJ, Kim BG, Bae DS, Lee JW. Anticancer Res. 2013 Apr;33(4):1555-61.
27. The inhibitory effect of salinomycin on the proliferation, migration and invasion of human endometrial cancer stem-like cells. Kusunoki S, Kato K, Tabu K, Inagaki T, Okabe H, Kaneda H, Suga S, Terao Y, Taga T, Takeda S. Gynecol Oncol. 2013 Jun;129(3):598-605.
28. The expression status of G protein-coupled receptor GPR30 is associated with the clinical characteristics of endometriosis. Yuguchi H, Tanabe A, Hayashi A, Tanaka Y, Okuda K, Yamashita Y, Terai Y, Ohmichi M. Endocr Res. 2013;38(4):223-31.
29. Effects of ectopic HER-2/neu gene expression on the COX-2/PGE2/P450arom signaling pathway in endometrial carcinoma cells: HER-2/neu gene expression in endometrial carcinoma cells. Li S, Ma X, Ma L, Wang C, He Y, Yu Z. J Exp Clin Cancer Res. 2013 Mar 2;32:11.
30. Aquaporin-1 plays a crucial role in estrogen-induced tubulogenesis of vascular endothelial cells. Zou LB, Shi S, Zhang RJ, Wang TT, Tan YJ, Zhang D, Fei XY, Ding GL, Gao Q, Chen C, Hu XL, Huang HF, Sheng JZ. J Clin Endocrinol Metab. 2013 Apr;98(4):E672-82.
31. Indoleamine 2,3-dioxygenase-1 (IDO1) enhances survival and invasiveness of endometrial stromal cells via the activation of JNK signaling pathway. Mei J, Li MQ, Ding D, Li DJ, Jin LP, Hu WG, Zhu XY. Int J Clin Exp Pathol. 2013;6(3):431-44.
32. BRAF V600E Mutations in Endometrial Adenocarcinoma. He M, Breese V, Hang S, Zhang C, Xiong J, Jackson C. Diagn Mol Pathol. 2013 Mar;22(1):35-40.
33. Expression of adiponectin receptor 1 is indicative of favorable prognosis in non-small cell lung carcinoma. Abdul-Ghafar J, Oh SS, Park SM, Wairagu P, Lee SN, Jeong Y, Eom M, Yong SJ, Jung SH. Tohoku J Exp Med. 2013;229(2):153-62.
34. Lack of estrogen receptor-α is associated with epithelial-mesenchymal transition and PI3K alterations in endometrial carcinoma. Wik E, Ræder MB, Krakstad C, Trovik J, Birkeland E, Hoivik EA, Mjos S, Werner HM, Mannelqvist M, Stefansson IM, Oyan AM, Kalland KH, Akslen LA, Salvesen HB. Clin Cancer Res. 2013 Mar 1;19(5):1094-105.
35. Expression of hPOT1 in HeLa cells and the probability of gene variation of hpot1 Exon14 in endometrial cancer are much higher than in other cancers. Liu F, Pu XY, Huang SG, Xiang GM, Jiang DN, Hou G, Huang DN. Asian Pac J Cancer Prev. 2012;13(11):5659-63.
36. Down-regulated expression of Notch signaling molecules in human endometrial cancer. Jonusiene V, Sasnauskiene A, Lachej N, Kanopiene D, Dabkeviciene D, Sasnauskiene S, Kazbariene B, Didziapetriene J. Med Oncol. 2013 Mar;30(1):438.
37. Fatty acid synthase is a potential therapeutic target in estrogen receptor-/progesterone receptor-positive endometrioid endometrial cancer. Rahman MT, Nakayama K, Ishikawa M, Rahman M, Katagiri H, Katagiri A, Ishibashi T, Iida K, Miyazaki K. Oncology. 2013;84(3):166-73. PMID:
38. A role for two-pore potassium (K2P) channels in endometrial epithelial function. Patel SK, Jackson L, Warren AY, Arya P, Shaw RW, Khan RN. J Cell Mol Med. 2013 Jan;17(1):134-46.
39. *[Angiogenesis related gene expression profiles of human endometrial endothelial cells isolated from tissue of endometrial cancer]. Du XL, Sheng XG, Yan CX, Liu T, Wang C, Song QQ, Li QS. Zhonghua Fu Chan Ke Za Zhi. 2012 Oct;47(10):756-63. Chinese.*
40. *[Preliminary investigation of the expression and functions of insulin receptor isoforms in endometrial carcinoma]. Zhang G, Li XP, Wang JL, Wei LH. Zhonghua Fu Chan Ke Za Zhi. 2012 Nov;47(11):839-45. Chinese.*
41. MicroRNA-138 suppresses neutrophil gelatinase-associated lipocalin expression and inhibits tumorigenicity. Lee YC, Tzeng WF, Chiou TJ, Chu ST. PLoS One. 2012;7(12):e52979.
42. Down-regulation of Sox7 is associated with aberrant activation of Wnt/b-catenin signaling in endometrial cancer. Chan DW, Mak CS, Leung TH, Chan KK, Ngan HY. Oncotarget. 2012 Dec;3(12):1546-56.
43. Dose dependent molecular effects of acrylamide and glycidamide in human cancer cell lines and human primary hepatocytes. Ehlers A, Lenze D, Broll H, Zagon J, Hummel M, Lampen A. Toxicol Lett. 2013 Feb 27;217(2):111-20.
44. **Correlation between VEGFR-2 receptor kinase domain-containing receptor (KDR) mRNA and angiotensin II receptor type 1 (AT1-R) mRNA in endometrial cancer. Piastowska-Ciesielska AW, Płuciennik E, Wójcik-Krowiranda K, Bieńkiewicz A, Nowakowska M, Pospiech K, Bednarek AK, Domińska K, Ochędalski T. Cytokine. 2013 Feb;61(2):639-44.**
45. Enhanced expression of G-protein coupled estrogen receptor (GPER/GPR30) in lung cancer. Jala VR, Radde BN, Haribabu B, Klinge CM. BMC Cancer. 2012 Dec 28;12:624.
46. MicroRNA-200b is overexpressed in endometrial adenocarcinomas and enhances MMP2 activity by downregulating TIMP2 in human endometrial cancer cell line HEC-1A cells. Dai Y, Xia W, Song T, Su X, Li J, Li S, Chen Y, Wang W, Ding H, Liu X, Li H, Zhao Q, Shao N. Nucleic Acid Ther. 2013 Feb;23(1):29-34.
47. **Altered expression of genes involved in progesterone biosynthesis, metabolism and action in endometrial cancer. Sinreih M, Hevir N, Rižner TL. Chem Biol Interact. 2013 Feb 25;202(1-3):210-7.**
48. Gene expression signatures differentiate uterine endometrial stromal sarcoma from leiomyosarcoma. Davidson B, Abeler VM, Hellesylt E, Holth A, Shih IeM, Skeie-Jensen T, Chen L, Yang Y, Wang TL. Gynecol Oncol. 2013 Feb;128(2):349-55.
49. **Prognostic value of tgfb1 protein in endometrioid adenocarcinoma. Soufla G, Sifakis S, Porichis F, Spandidos DA. Eur J Clin Invest. 2013 Jan;43(1):79-90.**
50. PEDF inhibits growth and invasiveness of endometrial cancer cells in vitro. Guo T, Gu C, Li B. Panminerva Med. 2012 Dec;54(4):299-304.
51. Altered claudin-4 expression in progesterone-treated endometrial adenocarcinoma cell line Ishikawa. Xiao-Yu P, Yan J, Cui-Ping F, Ya-Nan W, Hua L, Hua-Jun L. Int J Gynecol Cancer. 2012 Nov;22(9):1585-90.
52. The effects of DNA methylation and epigenetic factors on the expression of CD133 in ovarian cancers. Min KJ, So KA, Ouh YT, Hong JH, Lee JK. J Ovarian Res. 2012 Oct 15;5(1):28.
53. Comparison of endometrial regenerative cells and bone marrow stromal cells. [Wang H](http://www.ncbi.nlm.nih.gov/pubmed?term=Wang H%5BAuthor%5D&cauthor=true&cauthor_uid=23038994)^1^, [Jin P](http://www.ncbi.nlm.nih.gov/pubmed?term=Jin P%5BAuthor%5D&cauthor=true&cauthor_uid=23038994), [Sabatino M](http://www.ncbi.nlm.nih.gov/pubmed?term=Sabatino M%5BAuthor%5D&cauthor=true&cauthor_uid=23038994), [Ren J](http://www.ncbi.nlm.nih.gov/pubmed?term=Ren J%5BAuthor%5D&cauthor=true&cauthor_uid=23038994), [Civini S](http://www.ncbi.nlm.nih.gov/pubmed?term=Civini S%5BAuthor%5D&cauthor=true&cauthor_uid=23038994), [Bogin V](http://www.ncbi.nlm.nih.gov/pubmed?term=Bogin V%5BAuthor%5D&cauthor=true&cauthor_uid=23038994), [Ichim TE](http://www.ncbi.nlm.nih.gov/pubmed?term=Ichim TE%5BAuthor%5D&cauthor=true&cauthor_uid=23038994), [Stroncek DF](http://www.ncbi.nlm.nih.gov/pubmed?term=Stroncek DF%5BAuthor%5D&cauthor=true&cauthor_uid=23038994).J Transl Med 2012 Oct 5; 10:207.
54. Tyrosine kinase receptor status in endometrial stromal sarcoma: an immunohistochemical and genetic-molecular analysis. Cossu-Rocca P, Contini M, Uras MG, Muroni MR, Pili F, Carru C, Bosincu L, Massarelli G, Nogales FF, De Miglio MR. Int J Gynecol Pathol. 2012 Nov;31(6):570-9.
55. **Mammaglobin 1: not only a breast-specific and tumour-specific marker, but also a hormone-responsive endometrial protein. Classen-Linke I, Moss S, Gröting K, Beier HM, Alfer J, Krusche CA. Histopathology. 2012 Nov;61(5):955-65.**
56. Diagnostic and prognostic significance of miRNA signatures in tissues and plasma of endometrioid endometrial carcinoma patients. Torres A, Torres K, Pesci A, Ceccaroni M, Paszkowski T, Cassandrini P, Zamboni G, Maciejewski R. Int J Cancer. 2013 Apr 1;132(7):1633-45.
57. Molecular events in endometrial carcinosarcomas and the role of high mobility group AT-hook 2 in endometrial carcinogenesis. Romero-Pérez L, Castilla MÁ, López-García MÁ, Díaz-Martín J, Biscuola M, Ramiro-Fuentes S, Oliva E, Matias-Guiu X, Prat J, Cano A, Moreno-Bueno G, Palacios J. Hum Pathol. 2013 Feb;44(2):244-54.
58. Increased expression of the prolactin receptor is associated with malignant laryngeal tumors. González-Lucano LR, Muñoz-Valle JF, Ascencio-Cedillo R, Domínguez-Rosales JA, López-Rincón G, Del Toro-Arreola S, Bueno-Topete M, Daneri-Navarro A, Estrada-Chávez C, Pereira-Suárez AL. Exp Ther Med. 2012 Apr;3(4):603-607. Epub 2012 Jan 30.
59. Matrix metalloproteinase (MMP)-2 and MMP-9 expression in tumor infiltrating CD3 lymphocytes from women with endometrial cancer. Jedryka M, Chrobak A, Chelmonska-Soyta A, Gawron D, Halbersztadt A, Wojnar A, Kornafel J. Int J Gynecol Cancer. 2012 Oct;22(8):1303-9.
60. Mdm2 antagonists induce apoptosis and synergize with cisplatin overcoming chemoresistance in TP53 wild-type ovarian cancer cells. Mir R, Tortosa A, Martinez-Soler F, Vidal A, Condom E, Pérez-Perarnau A, Ruiz-Larroya T, Gil J, Giménez-Bonafé P. Int J Cancer. 2013 Apr 1;132(7):1525-36.
61. PIK3CA overexpression is a possible prognostic factor for favorable survival in ovarian clear cell carcinoma. Abe A, Minaguchi T, Ochi H, Onuki M, Okada S, Matsumoto K, Satoh T, Oki A, Yoshikawa H. Hum Pathol. 2013 Feb;44(2):199-207.
62. MicroRNA expression in ovarian carcinoma and its correlation with clinicopathological features. Lee H, Park CS, Deftereos G, Morihara J, Stern JE, Hawes SE, Swisher E, Kiviat NB, Feng Q. World J Surg Oncol. 2012 Aug 27;10:174.
63. CIP2A is overexpressed in human ovarian cancer and regulates cell proliferation and apoptosis. Fang Y, Li Z, Wang X, Zhang S. Tumour Biol. 2012 Dec;33(6):2299-306.
64. Deregulation of miR-100, miR-99a and miR-199b in tissues and plasma coexists with increased expression of mTOR kinase in endometrioid endometrial carcinoma. Torres A, Torres K, Pesci A, Ceccaroni M, Paszkowski T, Cassandrini P, Zamboni G, Maciejewski R. BMC Cancer. 2012 Aug 24;12:369.
65. A novel miR-193a-5p-YY1-APC regulatory axis in human endometrioid endometrial adenocarcinoma. Yang Y, Zhou L, Lu L, Wang L, Li X, Jiang P, Chan LK, Zhang T, Yu J, Kwong J, Cheung TH, Chung T, Mak K, Sun H, Wang H. Oncogene. 2013 Jul 18;32(29):3432-42.
66. Silencing of miR-148a in cancer-associated fibroblasts results in WNT10B-mediated stimulation of tumor cell motility. Aprelikova O, Palla J, Hibler B, Yu X, Greer YE, Yi M, Stephens R, Maxwell GL, Jazaeri A, Risinger JI, Rubin JS, Niederhuber J. Oncogene. 2013 Jul 4;32(27):3246-53.
67. *[Regulation of claudin-4 gene expression in endometrial adenocarcinoma Ishikawa cell line by progesterone]. Jin Y, Feng CP, Wang YN, Lin H, Li HJ, Pan XY. Zhonghua Fu Chan Ke Za Zhi. 2012 May;47(5):368-72. Chinese.*
68. **Enhanced expression of sulfatide, a sulfated glycolipid, in well-differentiated endometrial adenocarcinoma. Sugiyama T, Miyazawa M, Mikami M, Goto Y, Nishijima Y, Ikeda M, Hirasawa T, Muramatsu T, Takekoshi S, Iwamori M. Int J Gynecol Cancer. 2012 Sep;22(7):1192-7.**
69. microRNA-103 regulates the growth and invasion of endometrial cancer cells through the downregulation of tissue inhibitor of metalloproteinase 3. Yu D, Zhou H, Xun Q, Xu X, Ling J, Hu Y. Oncol Lett. 2012 Jun;3(6):1221-1226.
70. Expression of miRNAs and PTEN in endometrial specimens ranging from histologically normal to hyperplasia and endometrial adenocarcinoma. Lee H, Choi HJ, Kang CS, Lee HJ, Lee WS, Park CS. Mod Pathol. 2012 Nov;25(11):1508-15.
71. Plasma membrane proteomics identifies bone marrow stromal antigen 2 as a potential therapeutic target in endometrial cancer. Yokoyama T, Enomoto T, Serada S, Morimoto A, Matsuzaki S, Ueda Y, Yoshino K, Fujita M, Kyo S, Iwahori K, Fujimoto M, Kimura T, Naka T. Int J Cancer. 2013 Jan 15;132(2):472-84.
72. Insulin-like growth factors in endometrioid adenocarcinoma: correlation with clinico-pathological features and estrogen receptor expression. Liang YJ, Hao Q, Zhang HM, Wu YZ, Wang JD. BMC Cancer. 2012 Jun 21;12:262.
73. **Expression patterns of kinin-dependent genes in endometrial cancer. Orchel J, Witek L, Kimsa M, Strzalka-Mrozik B, Kimsa M, Olejek A, Mazurek U. Int J Gynecol Cancer. 2012 Jul;22(6):937-44.**
74. 5-aza-2'-deoxycytidine improves the sensitivity of endometrial cancer cells to progesterone therapy. Hu Q, Yu L, Chen R, Wang YL, Ji L, Zhang Y, Xie Y, Liao QP. Int J Gynecol Cancer. 2012 Jul;22(6):951-9.
75. Role of emmprin in endometrial cancer. Nakamura K, Kodama J, Hongo A, Hiramatsu Y. BMC Cancer. 2012 May 28;12:191.
76. **The expression of interleukin-8 and interleukin-8 receptors in endometrial carcinoma. Ewington L, Taylor A, Sriraksa R, Horimoto Y, Lam EW, El-Bahrawy MA. Cytokine. 2012 Aug;59(2):417-22.**
77. Somatostatin and somatostatin analogues reduce PDGF-induced endometrial cell proliferation and motility. Annunziata M, Luque RM, Durán-Prado M, Baragli A, Grande C, Volante M, Gahete MD, Deltetto F, Camanni M, Ghigo E, Castaño JP, Granata R. Hum Reprod. 2012 Jul;27(7):2117-29.
78. Endometrial miR-200c is altered during transformation into cancerous states and targets the expression of ZEBs, VEGFA, FLT1, IKKβ, KLF9, and FBLN5. Panda H, Pelakh L, Chuang TD, Luo X, Bukulmez O, Chegini N. Reprod Sci. 2012 Aug;19(8):786-96.
79. Role of the tripartite motif protein 27 in cancer development. Zoumpoulidou G, Broceño C, Li H, Bird D, Thomas G, Mittnacht S. J Natl Cancer Inst. 2012 Jun 20;104(12):941-52.
80. **Expression of the Wnt antagonist Dickkopf-3 is associated with prognostic clinicopathologic characteristics and impairs proliferation and invasion in endometrial cancer. Dellinger TH, Planutis K, Jandial DD, Eskander RN, Martinez ME, Zi X, Monk BJ, Holcombe RF. Gynecol Oncol. 2012 Aug;126(2):259-67.**
81. Identification of differentially expressed microRNAs in endometrial cancer cells after progesterone treatment. Bae J, Won M, Kim DY, Kim JH, Kim YM, Kim YT, Nam JH, Suh DS. Int J Gynecol Cancer. 2012 May;22(4):561-5.
82. Flavokawain B, a novel, naturally occurring chalcone, exhibits robust apoptotic effects and induces G2/M arrest of a uterine leiomyosarcoma cell line. Eskander RN, Randall LM, Sakai T, Guo Y, Hoang B, Zi X. J Obstet Gynaecol Res. 2012 Aug;38(8):1086-94.
83. **Somatic deletions of the polyA tract in the 3' untranslated region of epidermal growth factor receptor are common in microsatellite instability-high endometrial and colorectal carcinomas. Deqin M, Chen Z, Nero C, Patel KP, Daoud EM, Cheng H, Djordjevic B, Broaddus RR, Medeiros LJ, Rashid A, Luthra R. Arch Pathol Lab Med. 2012 May;136(5):510-6.**
84. Downregulation of membrane complement inhibitors CD55 and CD59 by siRNA sensitises uterine serous carcinoma overexpressing Her2/neu to complement and antibody-dependent cell cytotoxicity in vitro: implications for trastuzumab-based immunotherapy. Bellone S, Roque D, Cocco E, Gasparrini S, Bortolomai I, Buza N, Abu-Khalaf M, Silasi DA, Ratner E, Azodi M, Schwartz PE, Rutherford TJ, Pecorelli S, Santin AD. Br J Cancer. 2012 Apr 24;106(9):1543-50.
85. **Angiotensin II type I receptor and miR-155 in endometrial cancers: synergistic antiproliferative effects of anti-miR-155 and losartan on endometrial cancer cells. Choi CH, Park YA, Choi JJ, Song T, Song SY, Lee YY, Lee JW, Kim TJ, Kim BG, Bae DS. Gynecol Oncol. 2012 Jul;126(1):124-31.**
86. Prognostic significance of miR-205 in endometrial cancer. Karaayvaz M, Zhang C, Liang S, Shroyer KR, Ju J. PLoS One. 2012;7(4):e35158.
87. MicroRNA-125b down-regulation mediates endometrial cancer invasion by targeting ERBB2. Shang C, Lu YM, Meng LR. Med Sci Monit. 2012 Apr;18(4):BR149-55.
88. **Overexpression and ratio disruption of ΔNp63 and TAp63 isoform equilibrium in endometrial adenocarcinoma: correlation with obesity, menopause, and grade I/II tumors. Vakonaki E, Soulitzis N, Sifakis S, Papadogianni D, Koutroulakis D, Spandidos DA. J Cancer Res Clin Oncol. 2012 Aug;138(8):1271-8.**
89. Fibroblast growth factor receptor 2 gene amplification status and its clinicopathologic significance in gastric carcinoma. Jung EJ, Jung EJ, Min SY, Kim MA, Kim WH. Hum Pathol. 2012 Oct;43(10):1559-66.
90. Two novel GPER agonists induce gene expression changes and growth effects in cancer cells. Lappano R, Rosano C, Santolla MF, Pupo M, De Francesco EM, De Marco P, Ponassi M, Spallarossa A, Ranise A, Maggiolini M. Curr Cancer Drug Targets. 2012 Jun;12(5):531-42.
91. **Expression of genes encoding for enzymes associated with O-GlcNAcylation in endometrial carcinomas: clinicopathologic correlations. Krześlak A, Wójcik-Krowiranda K, Forma E, Bieńkiewicz A, Bryś M. Ginekol Pol. 2012 Jan;83(1):22-6.**
92. Effect of estrogen sulfation by SULT1E1 and PAPSS on the development of estrogen-dependent cancers. Xu Y, Liu X, Guo F, Ning Y, Zhi X, Wang X, Chen S, Yin L, Li X. Cancer Sci. 2012 Jun;103(6):1000-9.
93. **Overexpression of 17β-hydroxysteroid dehydrogenase type 1 increases the exposure of endometrial cancer to 17β-estradiol. Cornel KM, Kruitwagen RF, Delvoux B, Visconti L, Van de Vijver KK, Day JM, Van Gorp T, Hermans RJ, Dunselman GA, Romano A. J Clin Endocrinol Metab. 2012 Apr;97(4):E591-601.**
94. Reversed glucose and fatty acids transporter expression in human endometrial cancer. Knapp P, Chabowski A, Harasiuk D, Górski J. Horm Metab Res. 2012 Jun;44(6):436-41.
95. Expression of leukemia inhibitory factor in the endometrium in abnormal uterine cavities during the implantation window. Hasegawa E, Ito H, Hasegawa F, Hatano K, Kazuka M, Usuda S, Isaka K. Fertil Steril. 2012 Apr;97(4):953-8.
96. Polymorphism 1558 C &gt; T in the aromatase gene (CYP19A1) in low-grade endometrial stromal sarcoma. Reich O, Regauer S, Tempfer C, Schneeberger C, Huber J. Eur J Gynaecol Oncol. 2011;32(6):626-7.
97. Acrp30 inhibits leptin-induced metastasis by downregulating the JAK/STAT3 pathway via AMPK activation in aggressive SPEC-2 endometrial cancer cells. Wu X, Yan Q, Zhang Z, Du G, Wan X. Oncol Rep. 2012 May;27(5):1488-96.
98. Microparticles from ovarian carcinomas are shed into ascites and promote cell migration. Press JZ, Reyes M, Pitteri SJ, Pennil C, Garcia R, Goff BA, Hanash SM, Swisher EM. Int J Gynecol Cancer. 2012 May;22(4):546-52.
99. Deer (Cervus elaphus) antler extract suppresses adhesion and migration of endometriotic cells and regulates MMP-2 and MMP-9 expression. Kim JH, Yang YI, Ahn JH, Lee JG, Lee KT, Choi JH. J Ethnopharmacol. 2012 Mar 27;140(2):391-7.
100. Hypoxia upregulates ovarian cancer invasiveness via the binding of HIF-1α to a hypoxia-induced, methylation-free hypoxia response element of S100A4 gene. Horiuchi A, Hayashi T, Kikuchi N, Hayashi A, Fuseya C, Shiozawa T, Konishi I. Int J Cancer. 2012 Oct 15;131(8):1755-67.
101. **Expression of GLUT1 and GLUT3 glucose transporters in endometrial and breast cancers. Krzeslak A, Wojcik-Krowiranda K, Forma E, Jozwiak P, Romanowicz H, Bienkiewicz A, Brys M. Pathol Oncol Res. 2012 Jul;18(3):721-8.**
102. Identification of suitable reference genes for gene expression measurement in uterine sarcoma and carcinosarcoma tumors. Kowalewska M, Danska-Bidzinska A, Bakula-Zalewska E, Bidzinski M. Clin Biochem. 2012 Mar;45(4-5):368-71.
103. Metformin potentiates the effects of paclitaxel in endometrial cancer cells through inhibition of cell proliferation and modulation of the mTOR pathway. Hanna RK, Zhou C, Malloy KM, Sun L, Zhong Y, Gehrig PA, Bae-Jump VL. Gynecol Oncol. 2012 May;125(2):458-69.
104. Lim1/LIM1 is expressed in developing and adult mouse and human endometrium. Ye L, Evans J, Gargett CE. Histochem Cell Biol. 2012 Apr;137(4):527-36.
105. Effects of N-[N-(3, 5-difluorophenacetyl-L-alanyl)]-S-phenylglycine t-butyl ester (DAPT) on cell proliferation and apoptosis in Ishikawa endometrial cancer cells. Mori M, Miyamoto T, Yakushiji H, Ohno S, Miyake Y, Sakaguchi T, Hattori M, Hongo A, Nakaizumi A, Ueda M, Ohno E. Hum Cell. 2012 Mar;25(1):9-15.
106. Analysis of the expression of angiotensin II type 1 receptor and VEGF in endometrial adenocarcinoma with different clinicopathological characteristics. Piastowska-Ciesielska AW, Płuciennik E, Wójcik-Krowiranda K, Bieńkiewicz A, Bednarek A, Ochędalski T. Tumour Biol. 2012 Jun;33(3):767-74.
107. Role of circulating free alu DNA in endometrial cancer. Tanaka H, Tsuda H, Nishimura S, Nomura H, Kataoka F, Chiyoda T, Tanaka K, Iguchi Y, Susumu N, Aoki D. Int J Gynecol Cancer. 2012 Jan;22(1):82-6.
108. **Endoplasmic reticulum stress is activated in endometrial adenocarcinoma. Bifulco G, Miele C, Di Jeso B, Beguinot F, Nappi C, Di Carlo C, Capuozzo S, Terrazzano G, Insabato L, Ulianich L. Gynecol Oncol. 2012 Apr;125(1):220-5.**
109. microRNA-30c negatively regulates endometrial cancer cells by targeting metastasis-associated gene-1. Zhou H, Xu X, Xun Q, Yu D, Ling J, Guo F, Yan Y, Shi J, Hu Y. Oncol Rep. 2012 Mar;27(3):807-12.
110. Proliferation assays for estrogenicity testing with high predictive value for the in vivo uterotrophic effect. Wang S, Aarts JM, Evers NM, Peijnenburg AA, Rietjens IM, Bovee TF. J Steroid Biochem Mol Biol. 2012 Feb;128(3-5):98-106.
111. **Laser capture microdissection with genome-wide expression profiling displayed gene expression signatures in endometrioid endometrial cancer. Mokhtar NM, Ramzi NH, Yin-Ling W, Rose IM, Hatta Mohd Dali AZ, Jamal R. Cancer Invest. 2012 Feb;30(2):156-64.**
112. Biologically inhibitory effects of VEGF siRNA on endometrial carcinoma cells. Zhao S, Ma D, Dai H, Yuan F, Wang Z. Arch Gynecol Obstet. 2011 Dec;284(6):1533-41.
113. **Plasminogen activator inhibitor-1 (PAI-1) 4 G/5 G polymorphism and endometrial cancer. Influence of PAI-1 polymorphism on tissue PAI-1 antigen and mRNA expression and tumor severity. Gilabert-Estellés J, Ramón LA, Braza-Boïls A, Gilabert J, Chirivella M, España F, Estellés A. Thromb Res. 2012 Aug;130(2):242-7.**
114. MicroRNA-34b functions as a potential tumor suppressor in endometrial serous adenocarcinoma. Hiroki E, Suzuki F, Akahira J, Nagase S, Ito K, Sugawara J, Miki Y, Suzuki T, Sasano H, Yaegashi N. Int J Cancer. 2012 Aug 15;131(4):E395-404.
115. The microRNA-200 family is upregulated in endometrial carcinoma. Snowdon J, Zhang X, Childs T, Tron VA, Feilotter H. PLoS One. 2011;6(8):e22828.
116. Trop-2 overexpression in poorly differentiated endometrial endometrioid carcinoma: implications for immunotherapy with hRS7, a humanized anti-trop-2 monoclonal antibody. Bignotti E, Ravaggi A, Romani C, Falchetti M, Lonardi S, Facchetti F, Pecorelli S, Varughese J, Cocco E, Bellone S, Schwartz PE, Rutherford TJ, Santin AD. Int J Gynecol Cancer. 2011 Dec;21(9):1613-21.
117. The expression levels of stem cell markers importin13, c-kit, CD146, and telomerase are decreased in endometrial polyps. Hu J, Yuan R. Med Sci Monit. 2011 Aug;17(8):BR221-227.
118. **Expression and in vitro functions of the ghrelin axis in endometrial cancer. Fung JN, Seim I, Wang D, Obermair A, Chopin LK, Chen C. Horm Cancer. 2010 Oct;1(5):245-55.**
119. Progesterone receptor-B induction of BIRC3 protects endometrial cancer cells from AP1-59-mediated apoptosis. Neubauer NL, Ward EC, Patel P, Lu Z, Lee I, Blok LJ, Hanifi-Moghaddam P, Schink J, Kim JJ. Horm Cancer. 2011 Jun;2(3):170-81.
120. **Expression and prognostic value of WISP-1 in patients with endometrial endometrioid adenocarcinoma. Tang Q, Jiang X, Li H, Lin Z, Zhou X, Luo X, Liu L, Chen G. J Obstet Gynaecol Res. 2011 Jun;37(6):606-12.**
121. **cDNA microarray analysis and immunohistochemistry reveal a distinct molecular phenotype in serous endometrial cancer compared to endometrioid endometrial cancer. Chen Y, Yao Y, Zhang L, Li X, Wang Y, Zhao L, Wang J, Wang G, Shen D, Wei L, Zhao J. Exp Mol Pathol. 2011 Aug;91(1):373-84.**
122. Progesterone-regulated B4galnt2 expression is a requirement for embryo implantation in mice. Li PT, Liao CJ, Wu WG, Yu LC, Chu ST. Fertil Steril. 2011 Jun;95(7):2404-9, 2409.e1-3.
123. microRNA regulation of the expression of the estrogen receptor in endometrial cancer. Zhou J, Song T, Gong S, Zhong M, Su G. Mol Med Rep. 2010 May-Jun;3(3):387-92.
124. **Diagnostic and prognostic impact of serum HE4 detection in endometrial carcinoma patients. Bignotti E, Ragnoli M, Zanotti L, Calza S, Falchetti M, Lonardi S, Bergamelli S, Bandiera E, Tassi RA, Romani C, Todeschini P, Odicino FE, Facchetti F, Pecorelli S, Ravaggi A. Br J Cancer. 2011 Apr 26;104(9):1418-25.**
125. **Aberrant expression of the von Hippel-Lindau gene in human endometrial hyperplasia and endometrial carcinoma. Xu JY, Zhu WJ, Cao XZ, Li XF, Wu J. Int J Gynecol Cancer. 2011 Apr;21(3):430-4.**
126. Dysregulation of microRNA-204 mediates migration and invasion of endometrial cancer by regulating FOXC1. Chung TK, Lau TS, Cheung TH, Yim SF, Lo KW, Siu NS, Chan LK, Yu MY, Kwong J, Doran G, Barroilhet LM, Ng AS, Wong RR, Wang VW, Mok SC, Smith DI, Berkowitz RS, Wong YF. Int J Cancer. 2012 Mar 1;130(5):1036-45.
127. Up-regulation of microRNA-145 promotes differentiation by repressing OCT4 in human endometrial adenocarcinoma cells. Wu Y, Liu S, Xin H, Jiang J, Younglai E, Sun S, Wang H. Cancer. 2011 Sep 1;117(17):3989-98.
128. **Highly increased maspin expression corresponds with up-regulation of miR-21 in endometrial cancer: a preliminary report. Torres A, Torres K, Paszkowski T, Radej S, Staśkiewicz GJ, Ceccaroni M, Pesci A, Maciejewski R. Int J Gynecol Cancer. 2011 Jan;21(1):8-14.**
129. Decidual HtrA3 negatively regulates trophoblast invasion during human placentation. Singh H, Endo Y, Nie G. Hum Reprod. 2011 Apr;26(4):748-57.
130. Retinoic acid inhibits endometrial cancer cell growth via multiple genomic mechanisms. Cheng YH, Utsunomiya H, Pavone ME, Yin P, Bulun SE. J Mol Endocrinol. 2011 Mar 23;46(2):139-53.
131. Estrogen regulation of X-box binding protein-1 and its role in estrogen induced growth of breast and endometrial cancer cells. Sengupta S, Sharma CG, Jordan VC. Horm Mol Biol Clin Investig. 2010 Aug 1;2(2):235-243.
132. Metformin reverses progestin resistance in endometrial cancer cells by downregulating GloI expression. Zhang Z, Dong L, Sui L, Yang Y, Liu X, Yu Y, Zhu Y, Feng Y. Int J Gynecol Cancer. 2011 Feb;21(2):213-21.
133. Dysregulation of betaglycan expression in primary human endometrial carcinomas. Zakrzewski PK, Mokrosinski J, Cygankiewicz AI, Semczuk A, Rechberger T, Skomra D, Krajewska WM. Cancer Invest. 2011 Feb;29(2):137-44.
134. Uterine serous papillary carcinomas overexpress human trophoblast-cell-surface marker (Trop-2) and are highly sensitive to immunotherapy with hRS7, a humanized anti-Trop-2 monoclonal antibody. Varughese J, Cocco E, Bellone S, de Leon M, Bellone M, Todeschini P, Schwartz PE, Rutherford TJ, Pecorelli S, Santin AD. Cancer. 2011 Jul 15;117(14):3163-72.
135. Molecular markers of endometrial carcinoma detected in uterine aspirates. Colas E, Perez C, Cabrera S, Pedrola N, Monge M, Castellvi J, Eyzaguirre F, Gregorio J, Ruiz A, Llaurado M, Rigau M, Garcia M, Ertekin T, Montes M, Lopez-Lopez R, Carreras R, Xercavins J, Ortega A, Maes T, Rosell E, Doll A, Abal M, Reventos J, Gil-Moreno A. Int J Cancer. 2011 Nov 15;129(10):2435-44.
136. *[Expression and its clinical significance of hsa-miR-155 in serum of endometrial cancer]. Tan ZQ, Liu FX, Tang HL, Su Q. Zhonghua Fu Chan Ke Za Zhi. 2010 Oct;45(10):772-4. Chinese.*
137. The adult stem cell marker Musashi-1 modulates endometrial carcinoma cell cycle progression and apoptosis via Notch-1 and p21WAF1/CIP1. Götte M, Greve B, Kelsch R, Müller-Uthoff H, Weiss K, Kharabi Masouleh B, Sibrowski W, Kiesel L, Buchweitz O. Int J Cancer. 2011 Oct 15;129(8):2042-9.
138. Elevated oestrogen receptor splice variant ERαΔ5 expression in tumour-adjacent hormone-responsive tissue. Taylor SE, Patel II, Singh PB, Nicholson CM, Stringfellow HF, Gopala Krishna RK, Matanhelia SS, Martin-Hirsch PL, Martin FL. Int J Environ Res Public Health. 2010 Nov;7(11):3871-89.
139. Micro-RNA signature of the epithelial-mesenchymal transition in endometrial carcinosarcoma. Castilla MÁ, Moreno-Bueno G, Romero-Pérez L, Van De Vijver K, Biscuola M, López-García MÁ, Prat J, Matías-Guiu X, Cano A, Oliva E, Palacios J. J Pathol. 2011 Jan;223(1):72-80.
140. Overexpression of the hBiot2 gene is associated with development of human cervical cancer. Shen YM, He X, Deng HX, Xie YP, Wang CT, Wei YQ, Zhao X. Oncol Rep. 2011 Jan;25(1):75-80.
141. Loss of glutathione peroxidase 3 expression is correlated with epigenetic mechanisms in endometrial adenocarcinoma. Falck E, Karlsson S, Carlsson J, Helenius G, Karlsson M, Klinga-Levan K. Cancer Cell Int. 2010 Nov 24;10:46.
142. *[Regulation of Bub1 mRNA expression in endometrial carcinoma Ishikawa cells by estrogen and paclitaxel]. Chen YH, Li XP, Wang Y, Wang JL, Wei LH. Zhonghua Fu Chan Ke Za Zhi. 2010 Sep;45(9):686-90. Chinese.*
143. Endometrioid ovarian cancer and endometriotic cells exhibit the same alteration in the expression of interleukin-1 receptor II: to a link between endometriosis and endometrioid ovarian cancer. Keita M, AinMelk Y, Pelmus M, Bessette P, Aris A. J Obstet Gynaecol Res. 2011 Feb;37(2):99-107.
144. NSSR1 is regulated by testosterone in the mouse uterus and extensively expressed in endometrial carcinoma. Peng ZY, Xiao PJ, Qi Y, Zhang W, Chen XH, Xu P. Tumour Biol. 2011 Apr;32(2):359-66.
145. **Isomorph expression of BAG-1 gene, ER and PR in endometrial cancer. Porichi O, Nikolaidou ME, Apostolaki A, Arnogiannaki N, Papassideri I, Chatonidis I, Tserkezoglou A, Vorgias G, Kassanos D, Panotopoulou E. Anticancer Res. 2010 Oct;30(10):4103-8.**
146. **Gene expression profiling and cancer-related pathways in type I endometrial carcinoma. Saghir FS, Rose IM, Dali AZ, Shamsuddin Z, Jamal AR, Mokhtar NM. Int J Gynecol Cancer. 2010 Jul;20(5):724-31.**
147. **Disturbed expression of phase I and phase II estrogen-metabolizing enzymes in endometrial cancer: lower levels of CYP1B1 and increased expression of S-COMT. Hevir N, Sinkovec J, Rižner TL. Mol Cell Endocrinol. 2011 Jan 1;331(1):158-67.**
148. **Hyaluronan synthases (HAS1-3) and hyaluronidases (HYAL1-2) in the accumulation of hyaluronan in endometrioid endometrial carcinoma. Nykopp TK, Rilla K, Tammi MI, Tammi RH, Sironen R, Hämäläinen K, Kosma VM, Heinonen S, Anttila M. BMC Cancer. 2010 Sep 27;10:512.**
149. Rapamycin inhibits cell proliferation in type I and type II endometrial carcinomas: a search for biomarkers of sensitivity to treatment. Bae-Jump VL, Zhou C, Boggess JF, Whang YE, Barroilhet L, Gehrig PA. Gynecol Oncol. 2010 Dec;119(3):579-85.
150. The PI3K/Akt pathway upregulates Id1 and integrin α4 to enhance recruitment of human ovarian cancer endothelial progenitor cells. Su Y, Zheng L, Wang Q, Bao J, Cai Z, Liu A. BMC Cancer. 2010 Aug 26;10:459.
151. hI-con1, a factor VII-IgGFc chimeric protein targeting tissue factor for immunotherapy of uterine serous papillary carcinoma. Cocco E, Hu Z, Richter CE, Bellone S, Casagrande F, Bellone M, Todeschini P, Krikun G, Silasi DA, Azodi M, Schwartz PE, Rutherford TJ, Buza N, Pecorelli S, Lockwood CJ, Santin AD. Br J Cancer. 2010 Sep 7;103(6):812-9.
152. **Alternative splicing-related factor YT521: an independent prognostic factor in endometrial cancer. Zhang B, zur Hausen A, Orlowska-Volk M, Jäger M, Bettendorf H, Stamm S, Hirschfeld M, Yiqin O, Tong X, Gitsch G, Stickeler E. Int J Gynecol Cancer. 2010 May;20(4):492-9.**
153. Impact of the menstrual cycle on circulating cell-free DNA. Pölcher M, Ellinger J, Willems S, El-Maarri O, Höller T, Amann C, Wolfgarten M, Rudlowski C, Kuhn W, Braun M. Anticancer Res. 2010 Jun;30(6):2235-40.
154. Toll-like receptor (TLR) and nucleosome-binding oligomerization domain (NOD) gene polymorphisms and endometrial cancer risk. Ashton KA, Proietto A, Otton G, Symonds I, McEvoy M, Attia J, Scott RJ. BMC Cancer. 2010 Jul 21;10:382.
155. **Expression levels of hnRNP G and hTra2-beta1 correlate with opposite outcomes in endometrial cancer biology. Ouyang YQ, zur Hausen A, Orlowska-Volk M, Jäger M, Bettendorf H, Hirschfeld M, Tong XW, Stickeler E. Int J Cancer. 2011 May 1;128(9):2010-9.**
156. Significant association of genetic polymorphism of human nonmetastatic clone 23 type 1 gene with an increased risk of endometrial cancer. Wang PH, Yi YC, Tsai HT, Tee YT, Ko JL, Han CP, Liu YF, Lin LY, Yang SF. Gynecol Oncol. 2010 Oct;119(1):70-5.
157. Overexpressed epidermal growth factor receptor (EGFR)-induced progestin insensitivity in human endometrial carcinoma cells by the EGFR/mitogen-activated protein kinase signaling pathway. Ai Z, Wang J, Wang Y, Lu L, Tong J, Teng Y. Cancer. 2010 Aug 1;116(15):3603-13.
158. Impaired down-regulation of E-cadherin and beta-catenin protein expression in endometrial epithelial cells in the mid-secretory endometrium of infertile patients with endometriosis. Matsuzaki S, Darcha C, Maleysson E, Canis M, Mage G. J Clin Endocrinol Metab. 2010 Jul;95(7):3437-45.
159. Comprehensive miRNA profiling of surgically staged endometrial cancer. Cohn DE, Fabbri M, Valeri N, Alder H, Ivanov I, Liu CG, Croce CM, Resnick KE. Am J Obstet Gynecol. 2010 Jun;202(6):656.e1-8.
160. **Circulating estrogens in endometrial cancer cases and their relationship with tissular expression of key estrogen biosynthesis and metabolic pathways. Lépine J, Audet-Walsh E, Grégoire J, Têtu B, Plante M, Ménard V, Ayotte P, Brisson J, Caron P, Villeneuve L, Bélanger A, Guillemette C. J Clin Endocrinol Metab. 2010 Jun;95(6):2689-98.**
161. Expression of interleukin-1 (IL-1) ligands system in the most common endometriosis-associated ovarian cancer subtypes. Keita M, Bessette P, Pelmus M, Ainmelk Y, Aris A. J Ovarian Res. 2010 Jan 28;3:3.
162. Lysophosphatidic acid (LPA) effects on endometrial carcinoma in vitro proliferation, invasion, and matrix metalloproteinase activity. Wang FQ, Ariztia EV, Boyd LR, Horton FR, Smicun Y, Hetherington JA, Smith PJ, Fishman DA. Gynecol Oncol. 2010 Apr;117(1):88-95.
163. **Overexpression of EpCAM in uterine serous papillary carcinoma: implications for EpCAM-specific immunotherapy with human monoclonal antibody adecatumumab (MT201). El-Sahwi K, Bellone S, Cocco E, Casagrande F, Bellone M, Abu-Khalaf M, Buza N, Tavassoli FA, Hui P, Rüttinger D, Silasi DA, Azodi M, Schwartz PE, Rutherford TJ, Pecorelli S, Santin AD. Mol Cancer Ther. 2010 Jan;9(1):57-66.**
164. Prognostic value of tumour endothelial markers in patients with endometrial cancer. Bersinger NA, Schneider B, Vorburger SA, Johann S, Candinas D, Mueller MD. Oncol Lett. 2010 Jan;1(1):203-207. Epub 2010 Jan 1.
165. **Serum amyloid A: a novel biomarker for endometrial cancer. Cocco E, Bellone S, El-Sahwi K, Cargnelutti M, Buza N, Tavassoli FA, Schwartz PE, Rutherford TJ, Pecorelli S, Santin AD. Cancer. 2010 Feb 15;116(4):843-51.**
166. **Definition of microRNAs that repress expression of the tumor suppressor gene FOXO1 in endometrial cancer. Myatt SS, Wang J, Monteiro LJ, Christian M, Ho KK, Fusi L, Dina RE, Brosens JJ, Ghaem-Maghami S, Lam EW. Cancer Res. 2010 Jan 1;70(1):367-77.**
167. Notch1 expression correlates with tumor differentiation status in ovarian carcinoma. Wang M, Wang J, Wang L, Wu L, Xin X. Med Oncol. 2010 Dec;27(4):1329-35.
168. **Interleukin-11 in endometrial adenocarcinoma is regulated by prostaglandin F2alpha-F-prostanoid receptor interaction via the calcium-calcineurin-nuclear factor of activated T cells pathway and negatively regulated by the regulator of calcineurin-1. Sales KJ, Grant V, Cook IH, Maldonado-Pérez D, Anderson RA, Williams AR, Jabbour HN. Am J Pathol. 2010 Jan;176(1):435-45.**
169. **DcR1 expression in endometrial carcinomas. Tarragona J, Llecha N, Santacana M, Lopez S, Gatius S, Llobet D, Dolcet X, Palomar-Asenjo V, Gonzalez-Tallada FJ, Matias-Guiu X. Virchows Arch. 2010 Jan;456(1):39-44.**
170. In vitro activity of pertuzumab in combination with trastuzumab in uterine serous papillary adenocarcinoma. El-Sahwi K, Bellone S, Cocco E, Cargnelutti M, Casagrande F, Bellone M, Abu-Khalaf M, Buza N, Tavassoli FA, Hui P, Silasi DA, Azodi M, Schwartz PE, Rutherford TJ, Pecorelli S, Santin AD. Br J Cancer. 2010 Jan 5;102(1):134-43.
171. **BCL-2, BAX and P53 expression profiles in endometrial carcinoma as studied by real-time PCR and immunohistochemistry. Porichi O, Nikolaidou ME, Apostolaki A, Tserkezoglou A, Arnogiannaki N, Kassanos D, Margaritis L, Panotopoulou E. Anticancer Res. 2009 Oct;29(10):3977-82.**
172. **Gene expression profiling of endometrial adenocarcinomas reveals increased apolipoprotein E expression in poorly differentiated tumors. Huvila J, Brandt A, Rojas CR, Pasanen S, Talve L, Hirsimäki P, Fey V, Kytömäki L, Saukko P, Carpén O, Soini JT, Grénman S, Auranen A. Int J Gynecol Cancer. 2009 Oct;19(7):1226-31.**
173. Metformin is a potent inhibitor of endometrial cancer cell proliferation--implications for a novel treatment strategy. Cantrell LA, Zhou C, Mendivil A, Malloy KM, Gehrig PA, Bae-Jump VL. Gynecol Oncol. 2010 Jan;116(1):92-8.
174. Rapamycin potentiates the effects of paclitaxel in endometrial cancer cells through inhibition of cell proliferation and induction of apoptosis. Shafer A, Zhou C, Gehrig PA, Boggess JF, Bae-Jump VL. Int J Cancer. 2010 Mar 1;126(5):1144-54.
175. The AA genotype of a L1C G842A polymorphism is associated with an increased risk for ovarian cancer. Heubner M, Wimberger P, Kasimir-Bauer S, Otterbach F, Kimmig R, Siffert W. Anticancer Res. 2009 Aug;29(8):3449-52.
176. The molecular signature of endometriosis-associated endometrioid ovarian cancer differs significantly from endometriosis-independent endometrioid ovarian cancer. Banz C, Ungethuem U, Kuban RJ, Diedrich K, Lengyel E, Hornung D. Fertil Steril. 2010 Sep;94(4):1212-7.
177. Overexpression of epithelial cell adhesion molecule in primary, metastatic, and recurrent/chemotherapy-resistant epithelial ovarian cancer: implications for epithelial cell adhesion molecule-specific immunotherapy. Bellone S, Siegel ER, Cocco E, Cargnelutti M, Silasi DA, Azodi M, Schwartz PE, Rutherford TJ, Pecorelli S, Santin AD. Int J Gynecol Cancer. 2009 Jul;19(5):860-6.
178. **Serum amyloid A (SAA): a novel biomarker for uterine serous papillary cancer. Cocco E, Bellone S, El-Sahwi K, Cargnelutti M, Casagrande F, Buza N, Tavassoli FA, Siegel ER, Visintin I, Ratner E, Silasi DA, Azodi M, Schwartz PE, Rutherford TJ, Pecorelli S, Santin AD. Br J Cancer. 2009 Jul 21;101(2):335-41.**
179. Synuclein-gamma (SNCG) may be a novel prognostic biomarker in uterine papillary serous carcinoma. Morgan J, Hoekstra AV, Chapman-Davis E, Hardt JL, Kim JJ, Buttin BM. Gynecol Oncol. 2009 Aug;114(2):293-8.
180. Estrogen receptor polymorphisms and the risk of endometrial cancer.Ashton KA, Proietto A, Otton G, Symonds I, McEvoy M, Attia J, Gilbert M, Hamann U, Scott RJ. BJOG. 2009 Jul;116(8):1053-61.
181. Silencing of heat shock protein 70 expression enhances radiotherapy efficacy and inhibits cell invasion in endometrial cancer cell line. Du XL, Jiang T, Wen ZQ, Gao R, Cui M, Wang F. Croat Med J. 2009 Apr;50(2):143-50.
182. **P2X(7) receptor expression is decreased in epithelial cancer cells of ectodermal, uro-genital sinus, and distal paramesonephric duct origin. Li X, Qi X, Zhou L, Fu W, Abdul-Karim FW, Maclennan G, Gorodeski GI. Purinergic Signal. 2009 Sep;5(3):351-68.**
183. Junctional adhesion molecule A [corrected] expression in human endometrial carcinoma. Koshiba H, Hosokawa K, Kubo A, Tokumitsu N, Watanabe A, Honjo H. Int J Gynecol Cancer. 2009 Feb;19(2):208-13.
184. Genetic variants in MUTYH are not associated with endometrial cancer risk. Ashton KA, Proietto A, Otton G, Symonds I, Scott RJ. Hered Cancer Clin Pract. 2009 Jan 26;7(1):3.
185. 5-Aza-2'-deoxycytidine is a potent inhibitor of DNA methyltransferase 3B and induces apoptosis in human endometrial cancer cell lines with the up-regulation of hMLH1. Cui M, Wen Z, Chen J, Yang Z, Zhang H. Med Oncol. 2010 Jun;27(2):278-85.
186. TNFalpha gene silencing reduced lipopolysaccharide-promoted proliferation of endometriotic stromal cells. Miyamoto A, Taniguchi F, Tagashira Y, Watanabe A, Harada T, Terakawa N. Am J Reprod Immunol. 2009 Apr;61(4):277-85.
187. The reduction in pigment epithelium-derived factor is a sign of malignancy in ovarian cancer expressing low-level of vascular endothelial growth factor. Tsuchiya T, Nakahama K, Asakawa Y, Maemura T, Tanaka M, Takeda S, Morita M, Morita I. Gynecol Endocrinol. 2009 Feb;25(2):104-9.
188. Differential expression profiling of gene response to ionizing radiation in two endometrial cancer cell lines with distinct radiosensitivities. Du XL, Jiang T, Wen ZQ, Li QS, Gao R, Wang F. Oncol Rep. 2009 Mar;21(3):625-34.
189. Diagnostic and prognostic impact of osteopontin expression in endometrial cancer. Cho H, Kang ES, Kim YT, Kim JH. Cancer Invest. 2009 Mar;27(3):313-23.
190. Polymorphisms in TP53 and MDM2 combined are associated with high grade endometrial cancer. Ashton KA, Proietto A, Otton G, Symonds I, McEvoy M, Attia J, Gilbert M, Hamann U, Scott RJ. Gynecol Oncol. 2009 Apr;113(1):109-14.
191. Increased PADI4 expression in blood and tissues of patients with malignant tumors. Chang X, Han J, Pang L, Zhao Y, Yang Y, Shen Z. BMC Cancer. 2009 Jan 30;9:40.
192. Tamoxifen induces expression of immune response-related genes in cultured normal human mammary epithelial cells. Schild-Hay LJ, Leil TA, Divi RL, Olivero OA, Weston A, Poirier MC. Cancer Res. 2009 Feb 1;69(3):1150-5.
193. *[Polymorphism of glucose intolerance and insulin resistance susceptibility genes in oncological patients]. Ulybina IuM, Imianitov EN, Vasil'ev DA, Bershteĭn LM. Mol Biol (Mosk). 2008 Nov-Dec;42(6):947-56. Russian.*
194. Estrogen regulates DNA methyltransferase 3B expression in Ishikawa endometrial adenocarcinoma cells. Cui M, Wen Z, Yang Z, Chen J, Wang F. Mol Biol Rep. 2009 Nov;36(8):2201-7.
195. Expression profile of mammalian microRNAs in endometrioid adenocarcinoma. Wu W, Lin Z, Zhuang Z, Liang X. Eur J Cancer Prev. 2009 Feb;18(1):50-5.
196. Molecular classification of spontaneous endometrial adenocarcinomas in BDII rats. Samuelson E, Hedberg C, Nilsson S, Behboudi A. Endocr Relat Cancer. 2009 Mar;16(1):99-111.
197. Dysregulated microRNAs and their predicted targets associated with endometrioid endometrial adenocarcinoma in Hong Kong women. Chung TK, Cheung TH, Huen NY, Wong KW, Lo KW, Yim SF, Siu NS, Wong YM, Tsang PT, Pang MW, Yu MY, To KF, Mok SC, Wang VW, Li C, Cheung AY, Doran G, Birrer MJ, Smith DI, Wong YF. Int J Cancer. 2009 Mar 15;124(6):1358-65.
198. Expression of 17beta-hydroxysteroid dehydrogenases and other estrogen-metabolizing enzymes in different cancer cell lines. Smuc T, Rizner TL. Chem Biol Interact. 2009 Mar 16;178(1-3):228-33.
199. Gene alterations in tumor-associated endothelial cells from endometrial cancer. Du XL, Jiang T, Zhao WB, Wang F, Wang GL, Cui M, Wen ZQ. Int J Mol Med. 2008 Nov;22(5):619-32.
200. The influence of the Cyclin D1 870 G&gt;A polymorphism as an endometrial cancer risk factor. Ashton KA, Proietto A, Otton G, Symonds I, McEvoy M, Attia J, Gilbert M, Hamann U, Scott RJ. BMC Cancer. 2008 Sep 29;8:272.
201. Tumor suppressor effect of follistatin-like 1 in ovarian and endometrial carcinogenesis: a differential expression and functional analysis. Chan QK, Ngan HY, Ip PP, Liu VW, Xue WC, Cheung AN. Carcinogenesis. 2009 Jan;30(1):114-21.
202. Expression of adiponectin receptors, AdipoR1 and AdipoR2, in normal colon epithelium and colon cancer tissue. Yoneda K, Tomimoto A, Endo H, Iida H, Sugiyama M, Takahashi H, Mawatari H, Nozaki Y, Fujita K, Yoneda M, Inamori M, Nakajima N, Wada K, Nagashima Y, Nakagama H, Uozaki H, Fukayama M, Nakajima A. Oncol Rep. 2008 Sep;20(3):479-83.
203. Interleukin-10 attenuates TNF-alpha-induced interleukin-6 production in endometriotic stromal cells. Tagashira Y, Taniguchi F, Harada T, Ikeda A, Watanabe A, Terakawa N. Fertil Steril. 2009 May;91(5 Suppl):2185-92.
204. **Trefoil factor 3: a novel serum marker identified by gene expression profiling in high-grade endometrial carcinomas. Bignotti E, Ravaggi A, Tassi RA, Calza S, Rossi E, Falchetti M, Romani C, Bandiera E, Odicino FE, Pecorelli S, Santin AD. Br J Cancer. 2008 Sep 2;99(5):768-73.**
205. Submucosal uterine leiomyomas have a global effect on molecular determinants of endometrial receptivity. Rackow BW, Taylor HS. Fertil Steril. 2010 Apr;93(6):2027-34.
206. Quantifiable mRNA transcripts for tamoxifen-metabolising enzymes in human endometrium. Singh MN, Stringfellow HF, Walsh MJ, Ashton KM, Paraskevaidis E, Abdo KR, Martin-Hirsch PL, Phillips DH, Martin FL. Toxicology. 2008 Jul 10;249(1):85-90.
207. HOXA11 DNA methylation--a novel prognostic biomarker in ovarian cancer. Fiegl H, Windbichler G, Mueller-Holzner E, Goebel G, Lechner M, Jacobs IJ, Widschwendter M. Int J Cancer. 2008 Aug 1;123(3):725-9.
208. Global analysis of genes regulated by HOXA10 in decidualization reveals a role in cell proliferation. Lu Z, Hardt J, Kim JJ. Mol Hum Reprod. 2008 Jun;14(6):357-66.
209. **Analysis of the status of the novel estrogen receptor α (ERα) coactivator p72 in endometrial cancer and its cross talk with erbB-2 in the transactivation of ERα. Zhao L, Watanabe M, Yano T, Yanagisawa J, Nakagawa S, Oishi H, Wada-Hiraike O, Oda K, Minaguchi T, Yasugi T, Kato S, Taketani Y. Mol Med Rep. 2008 May-Jun;1(3):387-90.**
210. *[F10 gene knock-down mediated by RNA interference induces apoptosis of KLE cells]. Cui YG, Quan S, Xing FQ. Nan Fang Yi Ke Da Xue Xue Bao. 2008 Mar;28(3):317-9. Chinese.*
211. Imprinted tumor suppressor genes ARHI and PEG3 are the most frequently down-regulated in human ovarian cancers by loss of heterozygosity and promoter methylation. Feng W, Marquez RT, Lu Z, Liu J, Lu KH, Issa JP, Fishman DM, Yu Y, Bast RC Jr. Cancer. 2008 Apr 1;112(7):1489-502.
212. Wilms tumor gene protein 1 is associated with ovarian cancer metastasis and modulates cell invasion. Barbolina MV, Adley BP, Shea LD, Stack MS. Cancer. 2008 Apr 1;112(7):1632-41.
213. Expression of differentiation-associated gene icb-1 is estrogen-responsive in ovarian and breast cancer cell lines. Bollmann J, Ortmann O, Treeck O. J Steroid Biochem Mol Biol. 2008 Mar;109(1-2):16-21.
214. **Correlative expression of cyclooxygenase-1 (Cox-1) and human epidermal growth factor receptor type-2 (Her-2) in endometrial cancer. Sugimoto T, Koizumi T, Sudo T, Yamaguchi S, Kojima A, Kumagai S, Nishimura R. Kobe J Med Sci. 2007;53(5):177-87.**
215. Rapid genotyping using real-time fluorescent PCR of the Trp64Arg polymorphism of the beta3-adrenergic receptor gene and the -3826 A to G variant of the uncoupling protein-1 gene. Kikuchi A, Kuramoto Y, Noritake N, Murase H, Daimaru O, Nakakita T, Itoh S. Biochem Genet. 2007 Dec;45(11-12):769-73. Epub 2007 Nov 27. No abstract available.
216. Expression of human Biot2 and its potential function on carcinogenesis in endometrial cancer. Yangmei S, Xiang H, Hongxin D, Hanshuo Y, Feng P, Yuping X, Yuquan W, Xia Z. Acta Obstet Gynecol Scand. 2007;86(12):1503-9.
217. **Mammaglobin B expression in human endometrial cancer. Tassi RA, Bignotti E, Falchetti M, Calza S, Ravaggi A, Rossi E, Martinelli F, Bandiera E, Pecorelli S, Santin AD. Int J Gynecol Cancer. 2008 Sep-Oct;18(5):1090-6. Epub 2007 Nov 16.**
218. Disabled-1 is a large common fragile site gene, inactivated in multiple cancers. McAvoy S, Zhu Y, Perez DS, James CD, Smith DI. Genes Chromosomes Cancer. 2008 Feb;47(2):165-74.
219. Long, abundantly expressed non-coding transcripts are altered in cancer. Perez DS, Hoage TR, Pritchett JR, Ducharme-Smith AL, Halling ML, Ganapathiraju SC, Streng PS, Smith DI. Hum Mol Genet. 2008 Mar 1;17(5):642-55. Epub 2007 Nov 15.
220. **FGF2 transcript levels are positively correlated with EGF and IGF-1 in the malignant endometrium. Soufla G, Sifakis S, Spandidos DA. Cancer Lett. 2008 Feb 8;259(2):146-55. Epub 2007 Nov 19.**
221. Non-random inactivation of large common fragile site genes in different cancers. McAvoy S, Ganapathiraju SC, Ducharme-Smith AL, Pritchett JR, Kosari F, Perez DS, Zhu Y, James CD, Smith DI. Cytogenet Genome Res. 2007;118(2-4):260-9. Review.
222. Estradiol-17beta regulates vascular endothelial growth factor and Bcl-2 expression in HHUA cells. Zhi X, Honda K, Sumi T, Yasui T, Nobeyama H, Yoshida H, Ishiko O. Int J Oncol. 2007 Dec;31(6):1333-8.
223. Macrophage migration inhibitory factor up-regulates alpha(v)beta(3) integrin and vascular endothelial growth factor expression in endometrial adenocarcinoma cell line Ishikawa. Bondza PK, Metz CN, Akoum A. J Reprod Immunol. 2008 Apr;77(2):142-51. Epub 2007 Sep 12.
224. Class I histone deacetylase expression in the human cyclic endometrium and endometrial adenocarcinomas. Krusche CA, Vloet AJ, Classen-Linke I, von Rango U, Beier HM, Alfer J. Hum Reprod. 2007 Nov;22(11):2956-66. Epub 2007 Aug 29.
225. *[Overexpression of estrogen receptor-related receptor alpha can stimulate estrogen receptor negative endometrial cancer cell proliferation]. Sun PM, Wei LH, Gao M, Wang JL, Zhao LJ, Wang DP, Zhang JX. Zhonghua Fu Chan Ke Za Zhi. 2007 Jun;42(6):408-11. Chinese.*
226. Hypomethylation-induced expression of S100A4 in endometrial carcinoma. Xie R, Loose DS, Shipley GL, Xie S, Bassett RL Jr, Broaddus RR. Mod Pathol. 2007 Oct;20(10):1045-54. Epub 2007 Aug 3.
227. Trichostatin A, a histone deacetylase inhibitor, attenuates invasiveness and reactivates E-cadherin expression in immortalized endometriotic cells. Wu Y, Starzinski-Powitz A, Guo SW. Reprod Sci. 2007 May;14(4):374-82.
228. **Coexpression index of estrogen receptor alpha mRNA isoforms in simple, complex hyperplasia without atypia, complex atypical hyperplasia and adenocarcinoma. Witek A, Paul-Samojedny M, Stojko R, Seifert B, Mazurek U. Gynecol Oncol. 2007 Aug;106(2):407-12. Epub 2007 Jun 11.**
229. Decreased expression of P2X7 in endometrial epithelial pre-cancerous and cancer cells. Li X, Qi X, Zhou L, Catera D, Rote NS, Potashkin J, Abdul-Karim FW, Gorodeski GI. Gynecol Oncol. 2007 Jul;106(1):233-43. Epub 2007 May 4.
230. **HER-2/neu overexpression and amplification in uterine serous papillary carcinoma: comparative analysis of immunohistochemistry, real-time reverse transcription-polymerase chain reaction, and fluorescence in situ hybridization. Odicino FE, Bignotti E, Rossi E, Pasinetti B, Tassi RA, Donzelli C, Falchetti M, Fontana P, Grigolato PG, Pecorelli S. Int J Gynecol Cancer. 2008 Jan-Feb;18(1):14-21. Epub 2007 Apr 19.**
231. Comparison of nucleic acid targets prepared from total RNA or poly(A) RNA for DNA oligonucleotide microarray hybridization. Petersen K, Oyan AM, Rostad K, Olsen S, Bø TH, Salvesen HB, Gjertsen BT, Bruserud O, Halvorsen OJ, Akslen LA, Steen VM, Jonassen I, Kalland KH. Anal Biochem. 2007 Jul 1;366(1):46-58. Epub 2007 Mar 18.
232. **CASC2a gene is down-regulated in endometrial cancer. Baldinu P, Cossu A, Manca A, Satta MP, Sini MC, Palomba G, Dessole S, Cherchi P, Mara L, Tanda F, Palmieri G. Anticancer Res. 2007 Jan-Feb;27(1A):235-43.**
233. Plausible linkage of hypoxia-inducible factor (HIF) in uterine endometrial cancers. Fujimoto J, Sato E, Alam SM, Jahan I, Toyoki H, Hong BL, Sakaguchi H, Tamaya T. Oncology. 2006;71(1-2):95-101. Epub 2007 Mar 6.
234. Expression of claudin-3 and claudin-4 in normal, hyperplastic, and malignant endometrial tissue. Pan XY, Wang B, Che YC, Weng ZP, Dai HY, Peng W. Int J Gynecol Cancer. 2007 Jan-Feb;17(1):233-41.
235. Expression of protease activated receptor-2 related to angiogenesis in tumor advancement of uterine endometrial cancers. Jahan I, Fujimoto J, Alam SM, Sato E, Sakaguchi H, Tamaya T. Oncol Rep. 2007 Feb;17(2):345-50.
236. Overexpression of ephrinB2 and EphB4 in tumor advancement of uterine endometrial cancers. Alam SM, Fujimoto J, Jahan I, Sato E, Tamaya T. Ann Oncol. 2007 Mar;18(3):485-90.
237. **Identification of molecular markers and signaling pathway in endometrial cancer in Hong Kong Chinese women by genome-wide gene expression profiling. Wong YF, Cheung TH, Lo KW, Yim SF, Siu NS, Chan SC, Ho TW, Wong KW, Yu MY, Wang VW, Li C, Gardner GJ, Bonome T, Johnson WB, Smith DI, Chung TK, Birrer MJ. Oncogene. 2007 Mar 22;26(13):1971-82.**
238. **Progesterone receptor isoforms as a prognostic marker in human endometrial carcinoma. Saito S, Ito K, Nagase S, Suzuki T, Akahira J, Okamura K, Yaegashi N, Sasano H. Cancer Sci. 2006 Dec;97(12):1308-14.**
239. Patterns of p73 N-terminal isoform expression and p53 status have prognostic value in gynecological cancers. Becker K, Pancoska P, Concin N, Vanden Heuvel K, Slade N, Fischer M, Chalas E, Moll UM. Int J Oncol. 2006 Oct;29(4):889-902.
240. **Expression of the epidermal growth factor system in endometrioid endometrial cancer. Ejskjaer K, Sørensen BS, Poulsen SS, Forman A, Nexø E, Mogensen O. Gynecol Oncol. 2007 Jan;104(1):158-67.**
241. High expression of insulin-like growth factor binding protein-2 messenger RNA in epithelial ovarian cancers produces elevated preoperative serum levels. Lancaster JM, Sayer RA, Blanchette C, Calingaert B, Konidari I, Gray J, Schildkraut J, Schomberg DW, Marks JR, Berchuck A. Int J Gynecol Cancer. 2006 Jul-Aug;16(4):1529-35.
242. **The up-regulation profiles of p21WAF1/CIP1 and RUNX1/AML1 correlate with myometrial infiltration in endometrioid endometrial carcinoma. Planagumà J, Gonzalez M, Doll A, Monge M, Gil-Moreno A, Baró T, García A, Xercavins J, Alameda F, Abal M, Reventós J. Hum Pathol. 2006 Aug;37(8):1050-7.**
243. Peroxisome proliferator-activated receptor gamma and growth inhibition by its ligands in uterine endometrial carcinoma. Ota K, Ito K, Suzuki T, Saito S, Tamura M, Hayashi S, Okamura K, Sasano H, Yaegashi N. Clin Cancer Res. 2006 Jul 15;12(14 Pt 1):4200-8.
244. **Urocortin expression is downregulated in human endometrial carcinoma. Florio P, De Falco G, Leucci E, Torricelli M, Torres PB, Toti P, Dell'Anna A, Tiso E, Santopietro R, Leoncini L, Petraglia F. J Endocrinol. 2006 Jul;190(1):99-105.**
245. Differentially androgen-modulated genes in ovarian epithelial cells from BRCA mutation carriers and control patients predict ovarian cancer survival and disease progression. Motamed-Khorasani A, Jurisica I, Letarte M, Shaw PA, Parkes RK, Zhang X, Evangelou A, Rosen B, Murphy KJ, Brown TJ. Oncogene. 2007 Jan 11;26(2):198-214.
246. **Claudin 1 differentiates endometrioid and serous papillary endometrial adenocarcinoma. Sobel G, Németh J, Kiss A, Lotz G, Szabó I, Udvarhelyi N, Schaff Z, Páska C. Gynecol Oncol. 2006 Nov;103(2):591-8.**
247. Estrogen-receptor-dependent regulation of telomerase activity in human endometrial cancer cell lines. Boggess JF, Zhou C, Bae-Jump VL, Gehrig PA, Whang YE. Gynecol Oncol. 2006 Nov;103(2):417-24.
248. Overexpression of kallikrein 10 (hK10) in uterine serous papillary carcinomas. Santin AD, Diamandis EP, Bellone S, Marizzoni M, Bandiera E, Palmieri M, Papasakelariou C, Katsaros D, Burnett A, Pecorelli S.Am J Obstet Gynecol. 2006 May;194(5):1296-302.
249. Expression of adiponectin receptors and its possible implication in the human endometrium. Takemura Y, Osuga Y, Yamauchi T, Kobayashi M, Harada M, Hirata T, Morimoto C, Hirota Y, Yoshino O, Koga K, Yano T, Kadowaki T, Taketani Y. Endocrinology. 2006 Jul;147(7):3203-10.
250. The Association of the COMT V158M Polymorphism with Endometrial/Ovarian Cancer in HNPCC Families Adhering to the Amsterdam Criteria. Ashton KA, Meldrum CJ, McPhillips ML, Suchy J, Kurzawski G, Lubinski J, Scott RJ. Hered Cancer Clin Pract. 2006 May 15;4(2):94-102.
251. The PTEN tumor suppressor inhibits telomerase activity in endometrial cancer cells by decreasing hTERT mRNA levels. Zhou C, Bae-Jump VL, Whang YE, Gehrig PA, Boggess JF. Gynecol Oncol. 2006 May;101(2):305-10.
252. AKR1C1 and AKR1C3 may determine progesterone and estrogen ratios in endometrial cancer. Rizner TL, Smuc T, Rupreht R, Sinkovec J, Penning TM. Mol Cell Endocrinol. 2006 Mar 27;248(1-2):126-35.
253. Expression analysis of estrogen-metabolizing enzymes in human endometrial cancer. Smuc T, Rupreht R, Sinkovec J, Adamski J, Rizner TL. Mol Cell Endocrinol. 2006 Mar 27;248(1-2):114-7.
254. Clinical relevance of dominant-negative p73 isoforms for responsiveness to chemotherapy and survival in ovarian cancer: evidence for a crucial p53-p73 cross-talk in vivo. Concin N, Hofstetter G, Berger A, Gehmacher A, Reimer D, Watrowski R, Tong D, Schuster E, Hefler L, Heim K, Mueller-Holzner E, Marth C, Moll UM, Zeimet AG, Zeillinger R. Clin Cancer Res. 2005 Dec 1;11(23):8372-83.
255. **Identification of a novel estrogen-regulated gene, EIG121, induced by hormone replacement therapy and differentially expressed in type I and type II endometrial cancer. Deng L, Broaddus RR, McCampbell A, Shipley GL, Loose DS, Stancel GM, Pickar JH, Davies PJ.Clin Cancer Res. 2005 Dec 1;11(23):8258-64.**
256. **Gene regulation profiles by progesterone and dexamethasone in human endometrial cancer Ishikawa H cells. Davies S, Dai D, Pickett G, Leslie KK. Gynecol Oncol. 2006 Apr;101(1):62-70.**
257. **Disturbance of circadian gene expression in endometrial cancer: detection by real-time quantitative RT-PCR. Shih HC, Choo KB, Chang TJ, Yang MY, Shih MC, Yeh KT, Liu TC, Lin SF, Chang JG. Oncol Rep. 2005 Dec;14(6):1533-8.**
258. Expression of icb-1 gene is interferon-gamma inducible in breast and ovarian cancer cell lines and affects the IFN gamma-response of SK-OV-3 ovarian cancer cells. Treeck O, Kindzorra I, Pauser K, Treeck L, Ortmann O. Cytokine. 2005 Nov 3;32(3-4):137-42.
259. **Real-time quantitative RT-PCR detection of disseminated endometrial tumor cells in peripheral blood and lymph nodes using the LightCycler System. Ji XQ, Sato H, Tanaka H, Konishi Y, Fujimoto T, Takahashi O, Tanaka T. Gynecol Oncol. 2006 Feb;100(2):355-60.**
260. **Up-regulation of ERM/ETV5 correlates with the degree of myometrial infiltration in endometrioid endometrial carcinoma. Planagumà J, Abal M, Gil-Moreno A, Díaz-Fuertes M, Monge M, García A, Baró T, Xercavins J, Reventós J, Alameda F. J Pathol. 2005 Dec;207(4):422-9.**
261. **Gene expression profiles of serous, endometrioid, and clear cell subtypes of ovarian and endometrial cancer. Zorn KK, Bonome T, Gangi L, Chandramouli GV, Awtrey CS, Gardner GJ, Barrett JC, Boyd J, Birrer MJ. Clin Cancer Res. 2005 Sep 15;11(18):6422-30.**
262. **Targeted therapy with a cytotoxic somatostatin analog, AN-238, inhibits growth of human experimental endometrial carcinomas expressing multidrug resistance protein MDR-1. Engel JB, Schally AV, Halmos G, Baker B, Nagy A, Keller G. Cancer. 2005 Sep 15;104(6):1312-21.**
263. **Gene expression profiling reveals novel regulation by bisphenol-A in estrogen receptor-alpha-positive human cells. Singleton DW, Feng Y, Yang J, Puga A, Lee AV, Khan SA. Environ Res. 2006 Jan;100(1):86-92.**
264. Epigenetic-mediated upregulation of progesterone receptor B gene in endometrial cancer cell lines. Xiong Y, Dowdy SC, Gonzalez Bosquet J, Zhao Y, Eberhardt NL, Podratz KC, Jiang SW. Gynecol Oncol. 2005 Oct;99(1):135-41.
265. Biallelic methylation and silencing of paternally expressed gene 3 (PEG3) in gynecologic cancer cell lines. Dowdy SC, Gostout BS, Shridhar V, Wu X, Smith DI, Podratz KC, Jiang SW. Gynecol Oncol. 2005 Oct;99(1):126-34.
266. **Aberrant expression and mutations of TGF-beta receptor type II gene in endometrial cancer. Sakaguchi J, Kyo S, Kanaya T, Maida Y, Hashimoto M, Nakamura M, Yamada K, Inoue M. Gynecol Oncol. 2005 Sep;98(3):427-33.**
267. *[Regulation of genistein on the levels of ERalpha, ERbeta mRNA in uterine endometrial cancer cells]. Xue XO, Wei LH. Beijing Da Xue Xue Bao. 2005 Jun 18;37(3):278-80. Chinese.*
268. Expression patterns of hyaluronan, hyaluronan synthases and hyaluronidases indicate a role for hyaluronan in the progression of endometrial cancer. Paiva P, Van Damme MP, Tellbach M, Jones RL, Jobling T, Salamonsen LA. Gynecol Oncol. 2005 Aug;98(2):193-202.
269. The increase of mitochondrial DNA content in endometrial adenocarcinoma cells: a quantitative study using laser-captured microdissected tissues. Wang Y, Liu VW, Xue WC, Tsang PC, Cheung AN, Ngan HY. Gynecol Oncol. 2005 Jul;98(1):104-10.
270. **Oestrogen regulated gene expression in normal and malignant endometrial tissue. O'Toole SA, Dunn E, Sheppard BL, Sheils O, O'Leary JJ, Wuttke W, Seidlova-Wuttke D. Maturitas. 2005 Jun 16;51(2):187-98.**
271. High serum levels of interleukin-6 in endometrial carcinoma are associated with uterine serous papillary histology, a highly aggressive and chemotherapy-resistant variant of endometrial cancer. Bellone S, Watts K, Cane' S, Palmieri M, Cannon MJ, Burnett A, Roman JJ, Pecorelli S, Santin AD. Gynecol Oncol. 2005 Jul;98(1):92-8.
272. Discovery of epigenetically masked tumor suppressor genes in endometrial cancer. Takai N, Kawamata N, Walsh CS, Gery S, Desmond JC, Whittaker S, Said JW, Popoviciu LM, Jones PA, Miyakawa I, Koeffler HP. Mol Cancer Res. 2005 May;3(5):261-9.
273. Human kallikrein 6: a new potential serum biomarker for uterine serous papillary cancer. Santin AD, Diamandis EP, Bellone S, Soosaipillai A, Cane S, Palmieri M, Burnett A, Roman JJ, Pecorelli S. Clin Cancer Res. 2005 May 1;11(9):3320-5.
274. Human telomerase RNA as endogenous control in endometrial tissue. Paul-Samojedny M, Witek A, Samojedny A, Witkowska A, Wilczok T. Int J Gynecol Cancer. 2005 Mar-Apr;15(2):343-8.
275. **Abnormal expression of period 1 (PER1) in endometrial carcinoma. Yeh KT, Yang MY, Liu TC, Chen JC, Chan WL, Lin SF, Chang JG. J Pathol. 2005 May;206(1):111-20.**
276. Histone deacetylase inhibitors decrease DNA methyltransferase-3B messenger RNA stability and down-regulate de novo DNA methyltransferase activity in human endometrial cells. Xiong Y, Dowdy SC, Podratz KC, Jin F, Attewell JR, Eberhardt NL, Jiang SW. Cancer Res. 2005 Apr 1;65(7):2684-9.
277. Inhibition of growth of experimental human endometrial cancer by an antagonist of growth hormone-releasing hormone. Engel JB, Keller G, Schally AV, Toller GL, Groot K, Havt A, Armatis P, Zarandi M, Varga JL, Halmos G. J Clin Endocrinol Metab. 2005 Jun;90(6):3614-21.
278. *[MMP-26 mRNA and estrogen receptor alpha co-expression in normal and pathological endometrium]. Pilka R, Kudela M, Eriksson P, Casslén B. Ceska Gynekol. 2005 Jan;70(1):56-62. Czech.*
279. **Regulation of tumor invasion by HOXB13 gene overexpressed in human endometrial cancer. Zhao Y, Yamashita T, Ishikawa M. Oncol Rep. 2005 Apr;13(4):721-6.**
280. Opposite alterations of DNA methyltransferase gene expression in endometrioid and serous endometrial cancers. Xiong Y, Dowdy SC, Xue A, Shujuan J, Eberhardt NL, Podratz KC, Jiang SW. Gynecol Oncol. 2005 Mar;96(3):601-9.
281. Promoter methylation and differential expression of pi-class glutathione S-transferase in endometrial carcinoma. Chan QK, Khoo US, Chan KY, Ngan HY, Li SS, Chiu PM, Man LS, Ip PP, Xue WC, Cheung AN. J Mol Diagn. 2005 Feb;7(1):8-16.
282. Epigenetic and genetic alterations of p33ING1b in ovarian cancer. Shen DH, Chan KY, Khoo US, Ngan HY, Xue WC, Chiu PM, Ip P, Cheung AN. Carcinogenesis. 2005 Apr;26(4):855-63.
283. Up-regulation of DNA methyltransferase 3B expression in endometrial cancers. Jin F, Dowdy SC, Xiong Y, Eberhardt NL, Podratz KC, Jiang SW. Gynecol Oncol. 2005 Feb;96(2):531-8.
284. **Overexpression of the TGF-beta antagonist Smad7 in endometrial cancer. Dowdy SC, Mariani A, Reinholz MM, Keeney GL, Spelsberg TC, Podratz KC, Janknecht R. Gynecol Oncol. 2005 Feb;96(2):368-73.**
285. Dose-dependent insulin regulation of insulin-like growth factor binding protein-1 in human endometrial stromal cells is mediated by distinct signaling pathways. Lathi RB, Hess AP, Tulac S, Nayak NR, Conti M, Giudice LC. J Clin Endocrinol Metab. 2005 Mar;90(3):1599-606.
286. **A differential gene expression profile reveals overexpression of RUNX1/AML1 in invasive endometrioid carcinoma. Planagumà J, Díaz-Fuertes M, Gil-Moreno A, Abal M, Monge M, García A, Baró T, Thomson TM, Xercavins J, Alameda F, Reventós J. Cancer Res. 2004 Dec 15;64(24):8846-53.**
287. Endometrial K-ras mutations in postmenopausal breast cancer patients treated with adjuvant tamoxifen or toremifene. Wallén M, Tomás E, Visakorpi T, Holli K, Mäenpää J. Cancer Chemother Pharmacol. 2005 Apr;55(4):343-6.
288. AKT involvement in cisplatin chemoresistance of human uterine cancer cells. Gagnon V, Mathieu I, Sexton E, Leblanc K, Asselin E. Gynecol Oncol. 2004 Sep;94(3):785-95.
289. **Matrix metalloproteinase-26 (matrilysin-2) expression is high in endometrial hyperplasia and decreases with loss of histological differentiation in endometrial cancer. Pilka R, Norata GD, Domanski H, Andersson C, Hansson S, Eriksson P, Casslén B. Gynecol Oncol. 2004 Sep;94(3):661-70.**
290. Inverse correlation of secreted frizzled-related protein 4 and beta-catenin expression in endometrial stromal sarcomas. Hrzenjak A, Tippl M, Kremser ML, Strohmeier B, Guelly C, Neumeister D, Lax S, Moinfar F, Tabrizi AD, Isadi-Moud N, Zatloukal K, Denk H. J Pathol. 2004 Sep;204(1):19-27.
291. **Endometrial TIMP-4 mRNA is high at midcycle and in hyperplasia, but down-regulated in malignant tumours. Coordinated expression with MMP-26. Pilka R, Domanski H, Hansson S, Eriksson P, Casslén B. Mol Hum Reprod. 2004 Sep;10(9):641-50.**
292. **Evaluation of mRNA expression of estrogen receptor beta and its isoforms in human normal and neoplastic endometrium. Skrzypczak M, Bieche I, Szymczak S, Tozlu S, Lewandowski S, Girault I, Radwanska K, Szczylik C, Jakowicki JA, Lidereau R, Kaczmarek L. Int J Cancer. 2004 Jul 20;110(6):783-7.**
293. Human CYP1B1 is regulated by estradiol via estrogen receptor. Tsuchiya Y, Nakajima M, Kyo S, Kanaya T, Inoue M, Yokoi T. Cancer Res. 2004 May 1;64(9):3119-25.
294. Drastic decrease of progesterone receptor form B but not A mRNA reflects poor patient prognosis in endometrial cancers. Sakaguchi H, Fujimoto J, Hong BL, Nakagawa Y, Tamaya T. Gynecol Oncol. 2004 May;93(2):394-9.
295. Identification of a novel candidate gene, CASC2, in a region of common allelic loss at chromosome 10q26 in human endometrial cancer. Baldinu P, Cossu A, Manca A, Satta MP, Sini MC, Rozzo C, Dessole S, Cherchi P, Gianfrancesco F, Pintus A, Carboni A, Deiana A, Tanda F, Palmieri G. Hum Mutat. 2004 Apr;23(4):318-26.
296. Comparison of human papillomavirus DNA levels in gynecological cancers: implication for cancer development. Yang HJ, Liu VW, Tsang PC, Yip AM, Ng TY, Cheung AN, Ngan HY. Tumour Biol. 2003 Nov-Dec;24(6):310-6.
297. Tamoxifen-associated malignant endometrial tumors: pathologic features and expression of hormone receptors estrogen-alpha, estrogen-beta and progesterone; a case controlled study. Wilder JL, Shajahan S, Khattar NH, Wilder DM, Yin J, Rushing RS, Beaven R, Kaetzel C, Ueland FR, van Nagell JR, Kryscio RJ, Lele SM. Gynecol Oncol. 2004 Feb;92(2):553-8.
298. **Expression, localization, and signaling of prostaglandin F2 alpha receptor in human endometrial adenocarcinoma: regulation of proliferation by activation of the epidermal growth factor receptor and mitogen-activated protein kinase signaling pathways. Sales KJ, Milne SA, Williams AR, Anderson RA, Jabbour HN. J Clin Endocrinol Metab. 2004 Feb;89(2):986-93.**
299. **Expression of estrogen receptor coregulators in normal and malignant human endometrium. Kershah SM, Desouki MM, Koterba KL, Rowan BG. Gynecol Oncol. 2004 Jan;92(1):304-13.**
300. Germline truncating mutations in both MSH2 and BRCA2 in a single kindred. Thiffault I, Hamel N, Pal T, McVety S, Marcus VA, Farber D, Cowie S, Deschênes J, Meschino W, Odefrey F, Goldgar D, Graham T, Narod S, Watters AK, MacNamara E, Du Sart D, Chong G, Foulkes WD. Br J Cancer. 2004 Jan 26;90(2):483-91.
301. **Expression of uteroglobin and matrix metalloproteinase-9 genes in endometrial cancer: relationship to estrogen and progesterone receptor status. Cioppi F, Simi L, Luciani P, Petraglia F, Susini T, Cobellis L, Serio M, Maggi M, Peri A. Oncol Rep. 2004 Feb;11(2):427-33.**
302. Large scale validation of human N-myc downstream-regulated gene (NDRG)-1 expression in endometrium during the menstrual cycle. Malette B, Cherry E, Lagacé M, Bernard M, Gosselin D, Hugo P, Shazand K. Mol Hum Reprod. 2003 Nov;9(11):671-9.
303. Estrogen regulates Ah responsiveness in MCF-7 breast cancer cells. Spink DC, Katz BH, Hussain MM, Pentecost BT, Cao Z, Spink BC. Carcinogenesis. 2003 Dec;24(12):1941-50.
304. Indole-3-carbinol is a negative regulator of estrogen. Auborn KJ, Fan S, Rosen EM, Goodwin L, Chandraskaren A, Williams DE, Chen D, Carter TH. J Nutr. 2003 Jul;133(7 Suppl):2470S-2475S. Review.
305. **KAI1 metastasis suppressor protein is down-regulated during the progression of human endometrial cancer. Liu FS, Dong JT, Chen JT, Hsieh YT, Ho ES, Hung MJ, Lu CH, Chiou LC. Clin Cancer Res. 2003 Apr;9(4):1393-8.**
306. Cyclin A1 is highly expressed in aggressive testicular germ cell tumors. Müller-Tidow C, Diederichs S, Schrader MG, Vogt U, Miller K, Berdel WE, Serve H. Cancer Lett. 2003 Feb 10;190(1):89-95.
307. **Increased expression of human luteinizing hormone/human chorionic gonadotropin receptor mRNA in human endometrial cancer. Ji Q, Chen P, Aoyoma C, Liu P. Mol Cell Probes. 2002 Aug;16(4):269-75.**
308. **Correlation of survivin mRNA detection with histologic diagnosis in normal endometrium and endometrial carcinoma. Lehner R, Enomoto T, McGregor JA, Shroyer L, Haugen BR, Pugazhenthi U, Shroyer KR. Acta Obstet Gynecol Scand. 2002 Feb;81(2):162-7.**
309. Expression of telomerase genes as potential marker of neoplastic changes. Mazurek U, Witek A, Olejek A, Paul M, Skałba P, Wilczok T. Folia Histochem Cytobiol. 2001;39 Suppl 2:183-4.
310. **Quantitative analysis of telomerase hTERT mRNA and telomerase activity in endometrioid adenocarcinoma and in normal endometrium. Lehner R, Enomoto T, McGregor JA, Shroyer AL, Haugen BR, Pugazhenthi U, Shroyer KR. Gynecol Oncol. 2002 Jan;84(1):120-5.**
311. Detection of microsatellite instability by real time PCR and hybridization probe melting point analysis. Dietmaier W, Hofstädter F. Lab Invest. 2001 Oct;81(10):1453-6.
312. Expression of COX-2 and PGE synthase and synthesis of PGE(2)in endometrial adenocarcinoma: a possible autocrine/paracrine regulation of neoplastic cell function via EP2/EP4 receptors. Jabbour HN, Milne SA, Williams AR, Anderson RA, Boddy SC. Br J Cancer. 2001 Sep 28;85(7):1023-31.
313. **Quantitation of Fas and Fas ligand gene expression in human ovarian, cervical and endometrial carcinomas using real-time quantitative RT-PCR. Das H, Koizumi T, Sugimoto T, Chakraborty S, Ichimura T, Hasegawa K, Nishimura R. Br J Cancer. 2000 May;82(10):1682-8.**
