# Supplementary material for: Identification of Optimal Reference Genes for Gene Expression Normalization in a Wide Cohort of Endometrioid Endometrial Carcinoma Tissues
Source: PLoS One. 2014 Dec 4;9(12):e113781. doi: 10.1371/journal.pone.0113781 (PMC4256201; doi:10.1371/journal.pone.0113781)
Supplement: Text S2 — Algorithm for testing equivalence and R code examples. (DOC) [file pone.0113781.s002.doc]

**Text S2: Algorithm for testing equivalence and R code examples**

**Algorithm for testing equivalence**

1. Establish a reference interval around the value of the null hypothesis: for a ratio FDA recommends [0.8-1.25]
2. Compute the 1-2*α confidence intervals (usually α=0.05) for the ratio of the two means
3. If the computed 90% confidence interval lies entirely within the reference interval then the null hypothesis of difference between the population means can be rejected.
4. For an ANOVA setting the procedure can be repetead for all pairwise contrasts rejecting the null hypotheses if all pairwise hypotheses are rejected.

**Example R code**

library(equivalence)

### Simulate some data ###

set.seed(4545) # set the seed for reproducibility

### Simulate some log-normal data (two groups gene expression) with little difference in population means

Xl<-rlnorm(1000,0,0.5)

Yl<-rlnorm(1000,0.1,0.5)

### log tranform the data

X = log(Xl)

Y = log(Yl)

#### TOST with epsiolon +/- 0.2231

tost(X,Y,epsilon=0.2231)
